# Anti-inflammatory aromadendrane- and cadinane-type sesquiterpenoids from the South China Sea sponge *Acanthella cavernosa*

**DOI:** 10.3762/bjoc.18.91

**Published:** 2022-07-25

**Authors:** Shou-Mao Shen, Qing Yang, Yi Zang, Jia Li, Xueting Liu, Yue-Wei Guo

**Affiliations:** 1 School of Chinese Materia Medica, Nanjing University of Chinese Medicine, Nanjing 210023, China; 2 State Key Laboratory of Drug Research, Shanghai Institute of Materia Medica, Chinese Academy of Sciences, Shanghai 201203, China; 3 School of Pharmaceutical Science, Nanchang University, Nanchang 330006, China; 4 Shandong Laboratory of Yantai Drug Discovery, Bohai Rim Advanced Research Institute for Drug Discovery, Yantai, Shandong 264117, China; 5 Open Studio for Druggability Research of Marine Natural Products, Pilot National Laboratory for Marine Science and Technology, Qingdao, China; 6 State Key Laboratory of Bioreactor Engineering, East China University of Science and Technology, Shanghai 200237, China

**Keywords:** *Acanthella cavernosa*, anti-inflammatory, biosynthetic pathway, chiral separation, marine sponge, sesquiterpenoid

## Abstract

One new aromadendrane-type sesquiterpenoid, namely ximaocavernosin P [(+)-**1**], and three new cadinane-type sesquiterpenoids, namely (+)-maninsigin D [(+)-**4**], (+)- and (−)-ximaocavernosin Q [(+)- and (−)-**5**], together with five related known ones [**2**, **3**, (−)-**4**, **6**, and **7**], were isolated from the Hainan sponge *Acanthella cavernosa*. Compounds **4** and **5** were isolated as racemic forms, which were further separated to the corresponding enantiomers [(+)-**4**/(−)-**4** and (+)-**5**/(−)-**5**], respectively, by using chiral-phase HPLC. The structures of new compounds were elucidated by extensive spectroscopic analysis and comparison with the reported data. In addition, the absolute configuration of optically pure (+)-**1** and **2** were determined by time-dependent density functional theory/electronic circular dichroism (TDDFT-ECD) calculations or X-ray diffraction analysis. A plausible biosynthetic pathway of these sesquiterpenoids and their internal correlation were proposed and discussed. In an in vitro bioassay, (+)-aristolone (**3**) exhibited promising anti-inflammatory activity by the inhibition of LPS-induced TNF-α and CCL2 release in RAW 264.7 macrophages.

## Introduction

Marine sponges of the genus *Acanthella* (class Demospongiae, order Halichondrida, family Axinellidae) are one of the most common marine invertebrates natively distributed throughout tropical and subtropical regions of the Indo-Pacific Ocean, in particular, the South China Sea [1]. They are well-known producers of various nitrogenous sesquiterpenes and diterpenes with characteristic isocyano, isothiocyano, and formamido functionalities [2–5]. Many of these secondary metabolites merit further investigation due to their intriguing structural diversity and wide spectra of biological activities ranging from antifeedant, antifouling, and cytotoxic to antibiotic effects [3,5–6]. *Acanthella* sponges have thus attracted much attention from marine natural products chemists and pharmacologists. The title animal is the most chemically studied species among the *Acanthella* sponges. Till now, more than 100 secondary metabolites belonging to sesquiterpenoids and diterpenes [7–8], alkaloids [9], and steroids [10], have been isolated and characterized.

**Figure 1:**
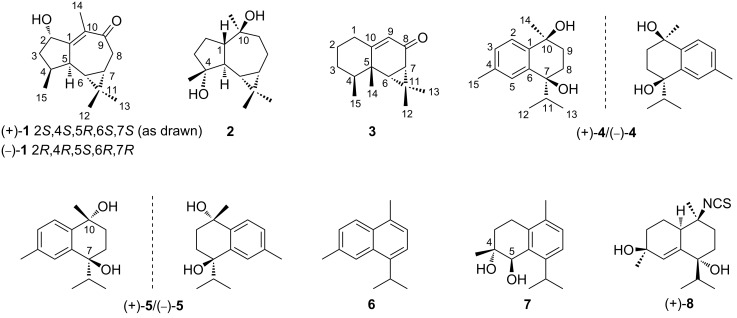
Chemical structures of compounds **1**–**8**.

In connection with our continuing studies of Chinese marine invertebrates to search for novel and bioactive secondary metabolites, we have recently studied the sponge *A. cavernosa* collected from Ximao Island of Hainan Province, China, resulting in the isolation of fifteen new nitrogenous sesquiterpenoids, exemplified by (+)-ximaocavernosin A (**8**) (Figure 1) [11]. To accumulate more amounts of these sesquiterpenoids to perform more in-depth pharmacological screening, we carried out the chemical investigation of the same sample on a large scale manner (310 g, dry weight). As a result, besides the already reported nitrogenous sesquiterpenoids, nine non-nitrogenous sesquiterpenoids, including a new aromadendrane-type sesquiterpenoid [(+)-**1**] and three new cadinane-type sesquiterpenoids [(+)-**4**, (+)-**5**, and (−)-**5**], together with five related known ones [**2**, **3**, (−)-**4**, **6**, and **7**] (Figure 1), were obtained. Herein, we report the isolation, chiral separation of racemic mixtures of **4** and **5**, structural elucidation, plausible biosynthetic pathway, and biological evaluation of these isolated compounds.

## Results and Discussion

By the similar workup [11] of the Et_2_O-soluble portion of the dichloromethane/methanol 1:1 extract of the title animal, five optically pure sesquiterpenoids [(+)-**1** (1.2 mg), **2** (4.1 mg), **3** (3.6 mg), **6** (1.3 mg), and **7** (0.5 mg)], as well as two non-optically pure compounds **4** (2.7 mg) and **5** (4.6 mg) were isolated.

**Figure 2:**
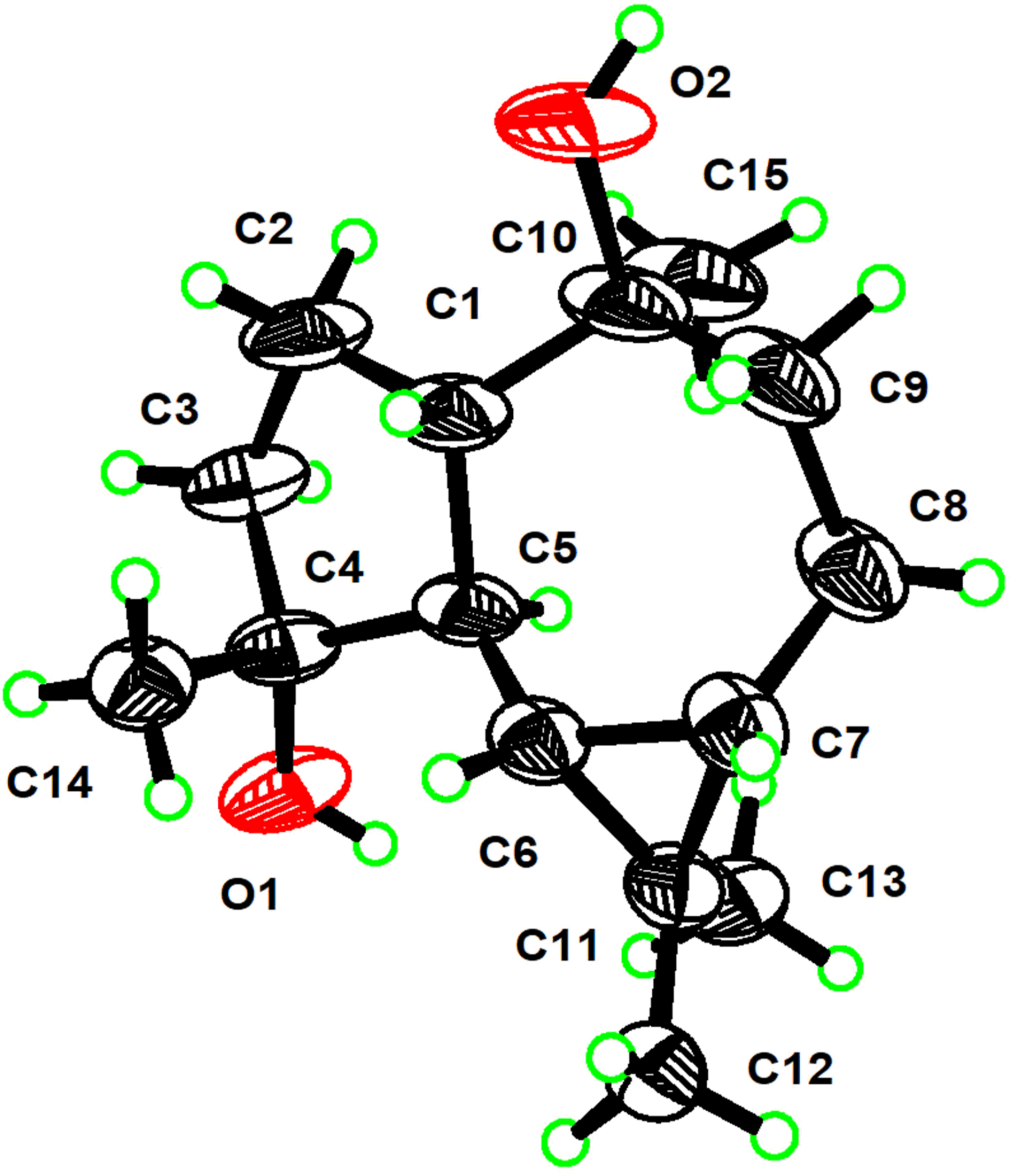
ORTEP drawing of **2** (displacement ellipsoids are drawn at the 50% probability level).

Among them, known compounds **2**, **3**, **6**, and **7** were readily identified as *ent*-4β,10α-dihydroxyaromadendrane (**2**) [12–14], (+)-aristolone (**3**) [15–16], cadalene (**6**) [17], and *trans*-4,5-dihydroxycorocalane (**7**) [18], respectively, by comparing their spectroscopic data and optical rotation values with those reported in the literature. In addition, the full structure of **2**, which was previously isolated from the soft coral *Sinularia mayi* [12], was unambiguously confirmed by X-ray diffraction analysis using Cu Kα radiation (λ = 1.54178 Å) [Flack parameter: 0.00 (11)] (Figure 2), since it was crystallized from MeCN at 4 °C in the present study.

Compound (+)-**1** was obtained as an optically active colorless oil. Its molecular formula was deduced to be C_15_H_22_O_2_ on the basis of HRESIMS [quasi-molecular ion peak at *m*/*z* 235.1700 ([M + H]^+^, calcd for 235.1693)]. The IR spectrum displayed an absorption band of an α,β-unsaturated ketone group (1644 cm^−1^), as additionally supported by the UV absorption at λ_max_ 245 nm (log ε 3.8). Careful analysis of the NMR spectra of (+)-**1** (Table 1 and Figures S8–S13 in Supporting Information File 1) showed a close similarity with those of co-occurring **2** [12–14], indicating compound (+)-**1** also being an aromadendrane-type sesquiterpenoid containing a *gem*-dimethylcyclopropyl unit [δ_H_ 0.80 (dd, *J* = 10.6, 9.6 Hz, H-6), 0.68 (m, H-7), 1.07 (s, 12-Me), 1.19 (s, 13-Me)]. Further literature survey revealed that the overall 1D and 2D NMR data of compound (+)-**1** (Table 1), except the optical rotation signal (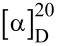 +169.6 (*c* 0.12, CHCl_3_) for (+)-**1**, 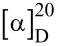 −186.0 (*c* 6.30, CHCl_3_) for (−)-**1**), were almost identical to those of 2β-hydroxyaromadendr-1(10)-en-9-one [(−)-**1**] [19], namely (1a*R*,5*R*,7*R*,7a*S*,7b*R*)-1,1a,2,5,6,7,7a,7b-octahydro-5-hydroxy-1,1,4,7-tetramethyl-3*H*-cycloprop[*e*]azulen-3-one, a biotransformation product of squamulosone [aromadendr-1(10)-en-9-one] by the fungus *Curvularia lunata* ATCC 12017 [19], indicating (+)-**1** and (−)-**1** were enantiomeric each other.

**Table 1:** ^1^H (*J* in Hz) and ^13^C NMR data of compounds (+)-**1**, **4**, and **5**.

no.	(+)-**1**		**4**			**5**		

	δ_H_^a,b^	δ_C_^a,c^	δ_H_^b,d^	δ_C_^c,d^	δ_C_^a,e^	δ_H_^b,d^	δ_C_^c,d^	δ_C_^a,e^

1	–	163.7 s	–	142.8 s	141.7	–	140.4 s	139.0
2	4.92 d (4.8)	74.5 d	7.47 d (8.4)	126.6 d	125.6	7.44 d (7.8)	127.2 d	125.8
3a	1.92 overlap	41.7 t	7.08 dd (8.4, 1.8)	129.2 d	129.0	7.08 dd (7.8, 1.8)	129.0 d	128.9
3b	1.56 dd (13.2, 4.8)	–	–	–	–	–	–	–
4	2.68 m	33.8 d	–	137.6 s	137.5	–	137.8 s	137.9
5	2.73 dd (9.6, 9.6)	43.7 d	7.28 brs	127.4 d	126.0	7.33 brs	127.9 d	126.7
6	0.80 dd (10.6, 9.6)	32.1 d	–	140.9 s	139.9	–	142.1 s	140.6
7	0.68 m	22.6 d	–	75.0 s	74.3	–	75.2 s	74.5
8a	2.38 dd (14.4, 11.4)	–	1.92 ddd (15.6, 10.8, 3.0)	28.4 t	27.9	2.26 ddd (13.8, 10.2, 3.0)	28.3 t	26.9
8b	2.84 dd (14.4, 4.8)	42.1 t	1.84 m	–	–	1.70 ddd (13.8, 8.0, 3.0)	–	–
9a	–	–	2.09 ddd (15.6, 10.8, 3.0)	35.6 t	35.6	2.00 ddd (13.8, 10.2, 3.0)	36.2 t	35.0
9b	–	201.9 s	1.81 m	–	–	1.92 ddd (13.8, 8.0, 1.2)	–	–
10	–	134.1 s	–	71.5 s	71.0	–	70.3 s	69.5
11	–	25.9 s	2.41 m	38.4 d	37.2	2.35 m	38.5 d	37.6
12	1.07 s	28.3 q	0.62 d (6.6)	16.5 q	16.2	0.71 d (6.6)	17.0 q	16.5
13	1.19 s	16.1 q	1.08 d (6.6)	18.9 q	18.7	1.08 d (6.6)	19.1 q	18.9
14	1.94 d (1.8)	14.8 q	1.40 s	30.2 q	29.7	1.54 s	31.2 q	30.6
15	1.05 d (6.6)	15.3 q	2.31 s	21.4 q	21.4	2.32 s	21.3 q	21.5

^a^Recorded in CDCl_3_, chemical shifts refer to CHCl_3_ (δ_H_ 7.26, δ_C_ 77.2); ^b^recorded at 600 MHz; ^c^recorded at 125 MHz; ^d^recorded in CD_3_OD, chemical shifts refer to CD_3_OD (δ_H_ 3.31, δ_C_ 49.0); ^e^recorded at 150 MHz.

**Figure 3:**
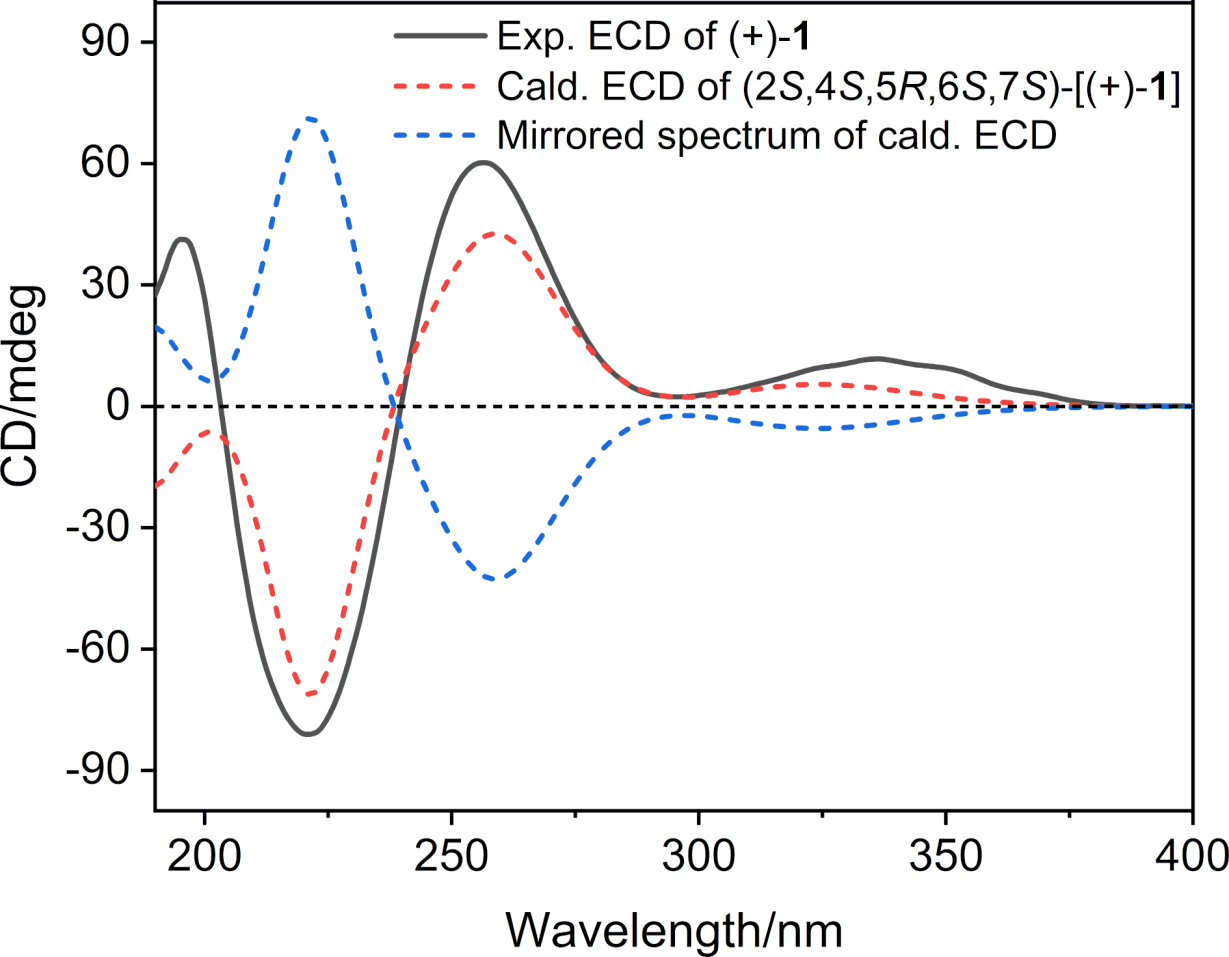
Experimental and calculated ECD curves of (+)-**1**.

To secure the absolute configuration of optically pure (+)-**1**, a TDDFT-ECD calculation, which has proven to be a reliable tool for the absolute configuration determination of natural products with stereogenic centers near the chromophore groups [20], was applied, since there is an α,β-unsaturated ketone chromophore nearby C-5 and C-2 in compound (+)-**1**. Thus, the theoretical ECD spectrum of (+)-**1** was calculated by the DFT calculation method at the b3lyp/6-311G** level of theory (see Supporting Information File 1 for details). A detailed comparison of the Boltzmann-averaged ECD spectrum with that of the experimental one (Figure 3) confirmed the absolute configuration of (+)-**1** as 2*S*, 4*S*, 5*R*, 6*S*, 7*S*. Consequently, the structure of (+)-**1** was assigned as depicted in Figure 1, and named (+)-ximaocavernosin P.

Compounds **4** and **5** showed NMR data diagnostic of tetrahydronaphthalene-bearing cadinane-type sesquiterpenoids, in line with the co-occurring compounds **6** and **7**. In addition, **4** and **5** as optically inactive white powder implied the possibility that both of them were present as racemic mixtures.

Compound **4** possessed a molecular formula of C_15_H_22_O_2_. Its structural elucidation was straightforward. The planar structure and relative configuration of **4** were immediately identified to be the same as those of (−)-maninsigin D [(−)-**4**] (Figure 1), a cadinane-type sesquiterpenoid of plant origin (leaves and stems of *Manglietia insignis*) [21], based on their identical ^1^H and ^13^C NMR data (Table 1 and Figures S16–S20 in Supporting Information File 1). Since the relative configuration of **4** (7S*, 10R*) was assigned, its absolute configuration was worth to be determined. However, the optical value at zero of **4** differed from that of (−)-maninsigin D [(−)-**4**: lit. 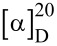 −9.4 (*c* 0.70, MeOH)] [21], suggesting that **4** should be present as a racemic mixture.

**Figure 4:**
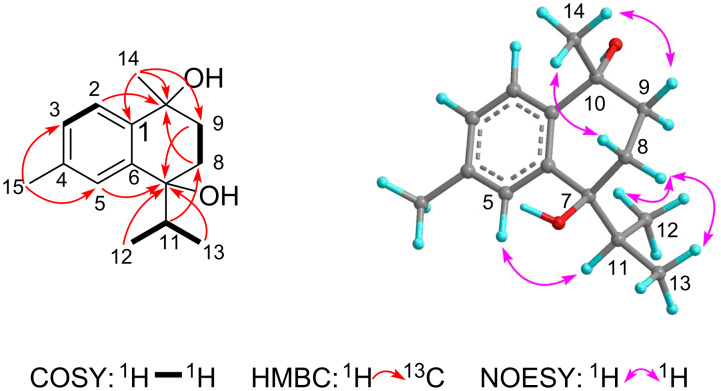
^1^H-^1^H COSY, key HMBC correlations, and NOESY correlations of compound **5**.

Ximaocavernosin Q (**5**) had the same molecular formula (C_15_H_22_O_2_) as **4**, implying compound **5** to be isomeric with **4**. The IR absorption band at 3386 cm^−1^ suggested the presence of hydroxy groups. A careful analysis of its 1D and 2D NMR spectra revealed that the NMR spectroscopic features of compound **5** (Table 1 and Figures S24–S31 in Supporting Information File 1) extremely resembled those of **4**. The main difference between them was obvious at C-10 and its neighboring carbons (e.g., C-1 and C-14). Considering the insignificant distinction between these chemical shift values (∆δ ≤ 2.4), compounds **4** and **5** were further analyzed using the same RP-HPLC conditions to examine whether they were the same compound (see Supporting Information File 1 for details). As a result, their distinct retention time [MeCN/H_2_O (35:65), 3.0 mL/min, 210 nm, **5**: *t*_R_ = 10.6 min, **4**: *t*_R_ = 17.6 min] (Figure S1, Supporting Information File 1) confirmed their different identities. Detailed analysis of their NMR spectroscopic data suggested an epimeric relationship between **5** and **4**. Epimerization at C-10 caused upfield shifts for C-10 and C-1 (δ_C_ 71.5 and 142.8 in **4**; δ_C_ 70.3 and 140.4 in **5**), whereas the resonance of C-14 was shifted downfield from δ_C_ 30.2 in **4** to δ_C_ 31.2 in **5**). Further, the diagnostic NOESY correlations between 14-Me (δ_H_ 1.54) and H-8b (δ_H_ 1.70)/H-9a (δ_H_ 2.00), between H-5 (δ_H_ 7.33) and H-11 (δ_H_ 2.35), as well as between H-8a (δ_H_ 2.26) and 12-Me (δ_H_ 0.71)/13-Me (δ_H_ 1.08), as shown in a computer-generated 3D drawing (Figure 4), determined the 7,10-*trans* relationship.

**Figure 5:**
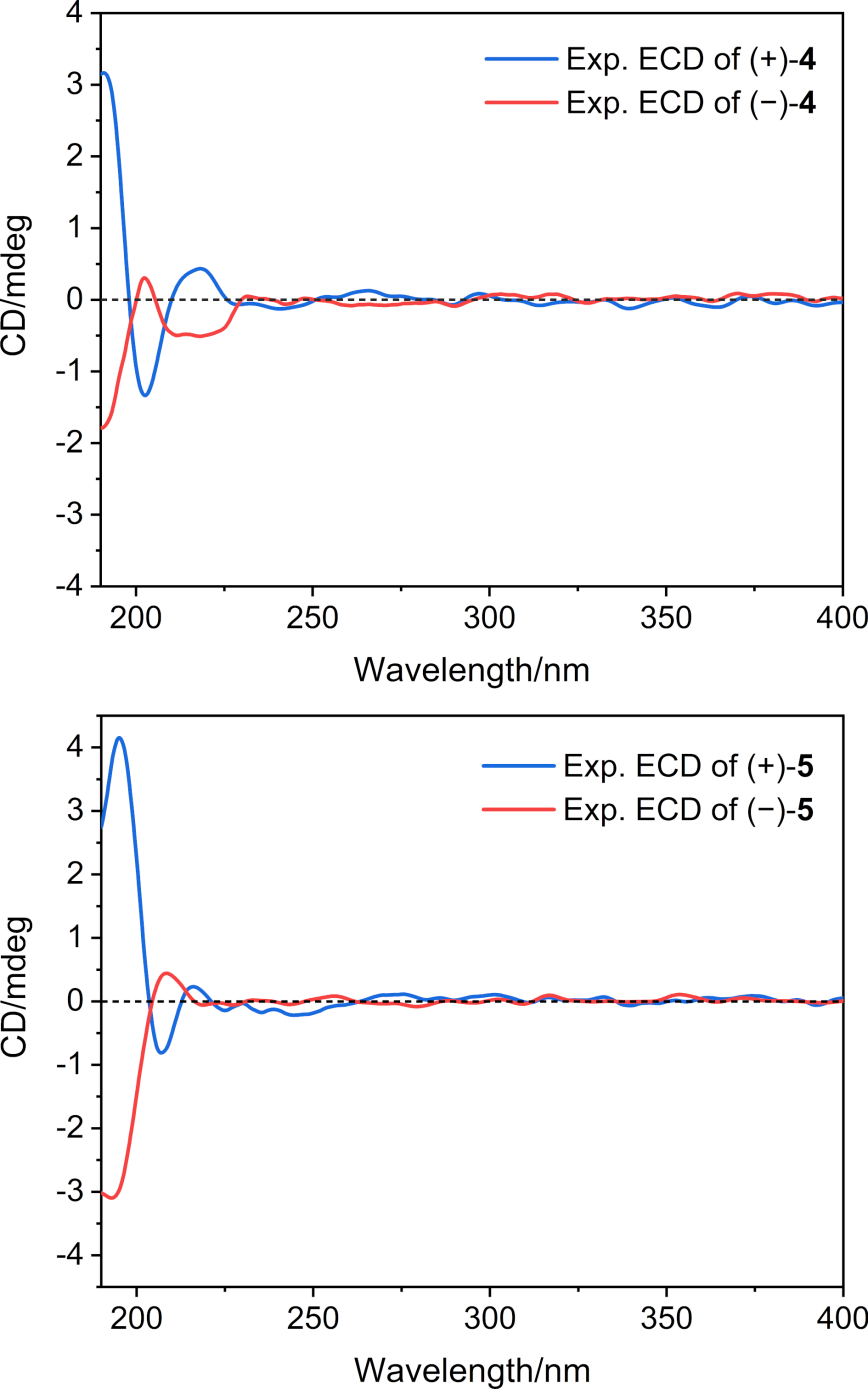
Experimental ECD curves of compounds (+)-**4**, (−)-**4** (top), (+)-**5**, and (−)-**5** (bottom).

To complete the full structure, the next step was to determine the stereochemistry at C-7 and C-10 of compounds **4** and **5**. Since both **4** and **5**, as mentioned above, are respective racemic mixtures, chiral-phase HPLC was then applied to separate each of them (see Figures S2 and S5 in Supporting Information File 1). As expected, new (+)-maninsigin D [(+)-**4**, 0.8 mg] and known (−)-maninsigin D [(−)-**4**, 0.8 mg] were afforded from **4** with opposite optical values [(+)-**4**: 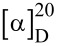 +26.0 (*c* 0.08, MeOH); (−)-**4**: 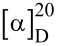 −24.2 (*c* 0.08, MeOH)] (Figures S3 and S4 in Supporting Information File 1) and the mirror image-like ECD curves (Figure 5). (+)-Ximaocavernosin Q [(+)-**5**, 0.8 mg] and (−)-ximaocavernosin Q [(−)-**5**, 0.7 mg] were obtained in the same way from **5** with 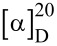 values of +8.8 (*c* 0.08, MeOH) and −8.3 (*c* 0.07, MeOH) (Figures S6 and S7 in Supporting Information File 1), and mirrored ECD curves (Figure 5). Having obtained two pairs of optically pure enantiomers [(+)-**4**/(−)-**4** and (+)-**5**/(−)-**5**], it is worth determining their absolute configuration. Unfortunately, our efforts to obtain suitable crystals for X-ray diffraction analysis were unsuccessful. The lack of secondary hydroxy groups in these molecules prevented the use of chemical approaches. In addition, their weak Cotton effects (Figure 5) also restricted the application of computational methods. Therefore, the absolute configuration for (+)-**4**/(−)-**4** and (+)-**5**/(−)-**5** remain undefined.

As mentioned in a review by William’s group [22], the enantiomeric natural products can arise from a single species or different genera and/or species. Several examples of enantiomers formation catalyzed by different terpene synthases were also reported. Jiang et al. characterized two new fungal bifunctional terpene synthases, FoFS and AtAS (identity 27.8%), that catalyzed the formation of a pair of enantiomeric sesterterpenes [23]. Two groups independently reported that the quiannulatene synthases EvQS [24] and AtTPS25 [25] (identity < 10%) from fungus and plant produced (+)- and (−)-quiannulatene, respectively. Interestingly, the cadinane-type sesquiterpenoids obtained from *A. cavernosa* generally exist in enantiomeric forms, such as our recently reported ximaocavernosins A–C, E, and G [11], as well as compounds **4** and **5** obtained in this present study. However, aromadendrane-type sesquiterpenoids from *A. cavernosa* are generally produced in optically pure forms, occasionally enantiomeric with others from a different origin, exemplified by (+)-ximaocavernosin P [(+)-**1**]. Further, these isothiocyano-containing enantiomers were usually isolated as scalable mixture (where one enantiomer predominates) with an enantiomeric excess of ca. 80% [11]. In contrast, the two neutral cadinane-type enantiomers (**4** and **5**) were produced as racemic mixture (1:1 ratio). Different enantiomeric ratios could explain the properties of the active sites in the corresponding terpene synthases, which remain unclear for further investigations [22].

**Scheme 1:**
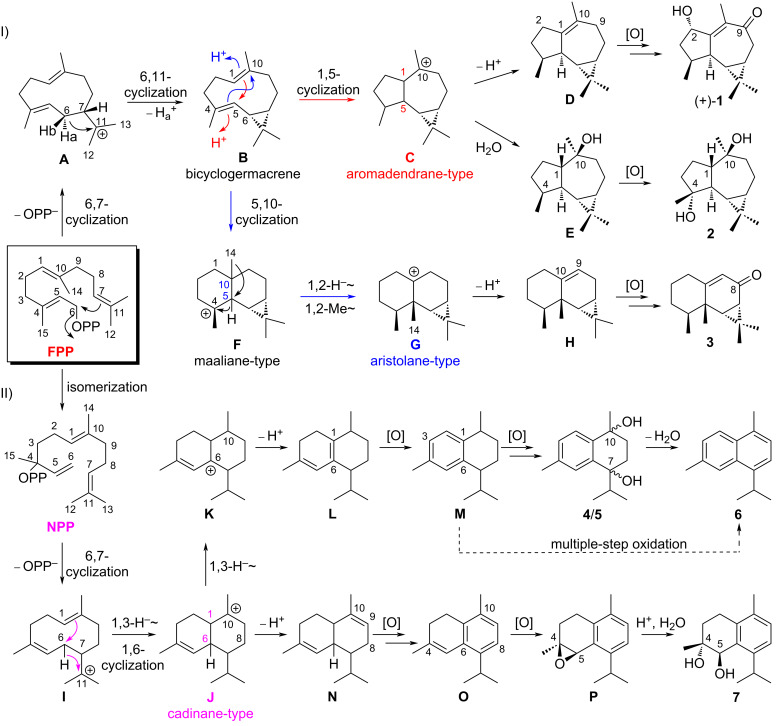
Proposed cyclization pathway of terpene intermediates and plausible post-modifications of compounds **1**–**7**.

The diversified structures of terpenes were constructed by terpene synthase [26] along with the post-modification enzymes, such as P450 enzymes and other oxygenases [27]. The plausible biosynthetic pathways of the isolated sesquiterpenoids **1**–**7** were proposed, as shown in Scheme 1. Aromadendrane- [(+)-**1** and **2**], aristolane- (**3**) and candinane-type sesquiterpenes [(+)-**4**/(−)-**4**, (+)-**5**/(−)-**5**, **6** and **7**)] were all originated from *E*,*E*-farnesyl diphosphate (*E*,*E*-FPP) as their linear precursor (Scheme 1).

The 6,7-bond formation was triggered by eliminating the pyrophosphate group of *E*,*E*-FPP yielding a monocyclic carbocation intermediate **A**, followed by the 6,11-closure via deprotonation to afford bicyclogermacrene (**B**) containing a *gem*-dimethylcyclopropyl unit [28]. Bicyclogermacrene (**B**) served as the branch point of aromadendrane cyclization and aristolane cyclization routes (Scheme 1, I). On one hand, the protonation on the double bond Δ^1,10^ of **B** initialized the aromadendrane cyclization, followed by 1,5-bond formation to yield carbocation intermediate **C**. Firstly, deprotonation occurred to form the double bond Δ^1,10^ and led to the formation of (−)-ledene (**D**) [29], on which the multiple-step oxidation happened at C-2 and C-9 to generate (+)-**1**. Secondly, the reaction is quenched by an H_2_O attach at carbocation C-10 to form (+)-globulol (**E**) [30], which was further oxidized to afford **2**. On the other hand, the aristolane cyclization route is started from protonation on Δ^4,5^ of **B** and followed by 5,10-cyclization to give maaliane-type carbocation intermediate **F**. Then, a successive transformation involving the concerted 1,2-hydride and 1,2-methyl shifts led to aristolane-type carbocation intermediate **G**, which was further deprotonated to afford 9-aristolene (**H**) [29]. Multiple-step oxidation on **H** furnished the structure of **3**.

The terpene cyclase catalyzed the cyclization of cadinene-type sesquiterpenes using FPP as the substrate, which is first isomerized to nerolidyl diphosphate (NPP), followed by the 6,7-bond formation to generate carbocation intermediate **I** (Scheme 1, II) [31]. Sequential 1,3-hydride shift and 1,6-cyclization occurred to afford cadinyl cation (**J**). Further 1,3-hydride shift and deprotonation on **J** resulted in cadina-1(6),4-diene (**L**) [32], the terpene precursor of compounds **4**–**6**. Meanwhile, direct deprotonation of cadinyl cation (**J**) generates the double bond Δ^9,10^ of 1,5-cadinadiene (**N**) [32], the precursor of compound **7**. Compounds **4**–**7** belong to the phenolic sesquiterpenes family, and the biosynthesis of the phenolic group has not yet been discovered. Phenolic sesquiterpenoids, for instance, illudacetalic acid and illudinine from *Omphalotus olearius*, were also discovered. Based on bioinformatics analysis, their aromatic rings were proposed to be constructed by putative P450 enzymes or oxidoreductase [33]. The Huang group characterized the function of a P450 enzyme CYP76AH1 which was responsible for the formation of the aromatic ring of ferruginol in the biosynthesis pathway of tanshinones [34]. Hence, we proposed that the oxidation occurred on **L** to furnish the aromatic ring of calamenene (**M**) [29], followed by the hydroxylation at C-7 and C-10 to give a pair of dihydroxy epimers **4** and **5**. Compound **6** could be generated by the dehydration of **4**/**5** or by the multiple-step oxidation of **M**. Similarly, the plausible biosynthetic pathway of compound **7** could be proposed below. Multiple-step oxidation of **N** gave a phenolic sesquiterpene α-corocalene (**O**) [35]. An epoxidation at C-4/C-5 of **O** resulted in the formation of α-corocalene epoxide (**P**) [35], which was further hydrolyzed to generate the final product **7** [36].

**Figure 6:**
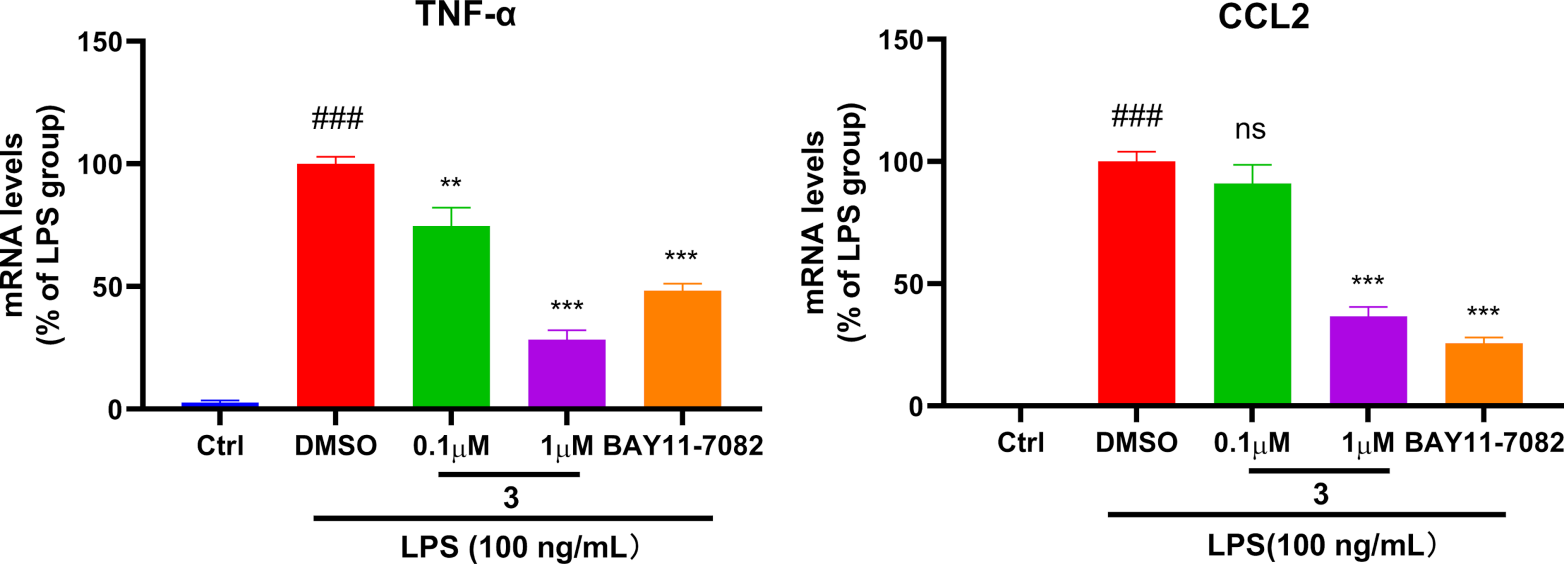
Compound **3** reduced the mRNA levels of TNF-α (left) and CCL2 (right) in LPS-stimulated RAW264.7 macrophages. Data were normalized by DMSO group and are presented as means ± standard errors of the mean (*n* = 3). ^###^*p* < 0.001 vs the control group; ^***^p < 0.001 vs the DMSO group.

In the anti-inflammatory assay, the transcriptional expression level of the representative inflammatory genes such as tumor necrosis factor-α (TNF-α) and C–C motif chemokine ligand 2 (CCL2) were investigated in lipopolysaccharide (LPS)-stimulated RAW 264.7 macrophages, using NF-κB inhibitor BAY-11-7082 as the positive control. Compound **3** displayed promising dose-dependent anti-inflammatory activity with the inhibition ratios of 74.1% in TNF-α and 64.1% in CCL2 at a concentration of 1 μM (Figure 6).

## Conclusion

The continuous chemical investigation of the sponge *A. cavernosa* has resulted in the isolation of nine non-nitrogenous sesquiterpenoids, of which four optically pure compounds [(+)-**1**, (+)-**4,** (+)-**5**, and (−)-**5**] are new. Compounds (+)-**1**, **2**, and **3** share a common *gem*-dimethylcyclopropyl unit, belonging to aromadendrane- and aristolane-type sesquiterpenoids, respectively. Whereas compounds **4**–**7** are a small group of cadinane-type sesquiterpenoids bearing a tetrahydronaphthalene system, which are produced in nature from isoprene units rather than by the much more common routes to aromatics involving acetate or shikimate [37]. In addition, the absolute stereochemistry of (+)-**1** was unambiguously elucidated by TDDFT-ECD calculations, and the complete structure of **2** was confirmed for the first time by X-ray diffraction analysis using Cu Kα radiation. Two pairs of racemic cadinane-type sesquiterpenoids (**4** and **5**) were successfully separated, by chiral-phase HPLC, to their corresponding enantiomers [(+)-**4**/(−)-**4** and (+)-**5**/(−)-**5**]. However, the determination of the absolute configuration of (+)-**4**/(−)-**4** and (+)-**5**/(−)-**5** requires further studies.

Notably, to our best knowledge, although a series of nitrogenous sesquiterpenoids from the genus *Acanthella* have been well documented in literature, less than six non-nitrogenous sesquiterpenoids have been reported from the title sponge [38–40]. The discovery of these sesquiterpenoids not only extended the members of the sesquiterpenoid family but also enriched the chemical diversity of the *Acanthella* sponges. The plausible biosynthetic pathway of isolated compounds **1**–**7** was also proposed. Up to date, no sesquiterpene cyclase has been characterized for aromadendrane and aristolane cyclization. The ubiquitous co-existence of enantiomeric cadinane-type sesquiterpenoids in the sponge revealed the unique catalytic properties of sponge-derived terpene synthases, which attracts our attention for further investigation of their structure–function relationship. Further elucidation of the cyclization mechanism and characterization of post-modification enzymes for the biosynthetic pathway of these sesquiterpenoids will provide insights into expanding the chemical space from marine sponges.

## Experimental

**General experimental procedure**. The melting point was recorded using an SGW X-4 micro-melting point apparatus (Shanghai Precision Scientific Apparatuses Co., Ltd, Shanghai, China). Optical rotations were measured on a Perkin-Elmer 241MC polarimeter (PerkinElmer, Fremont, CA, USA). IR spectra were recorded on a Nicolet 6700 spectrometer (Thermo Scientific, Waltham, MA, USA). ECD and UV spectra were measured with a JASCO J-815 instrument (JASCO, Japan). NMR spectra were measured on Bruker Avance III 500 and Bruker Avance III 600 instruments (Bruker Biospin AG, Rheinstetten, Germany) using TMS as an internal standard. Chemical shifts (δ) were reported with reference to the solvent signals, and coupling constants (*J*) were in Hz. HRESIMS spectra were recorded on an Agilent G6520 Q-TOF mass spectrometer, while HREIMS spectra were recorded on a Finnigan-MAT-95 mass spectrometer (Thermo Fisher Scientific, Waltham, USA). Commercial silica gel (Qingdao Haiyang Chemical Group Co., Ltd., Qingdao, China, 200–300 and 300–400 mesh), Sephadex LH-20 gel (Amersham Biosciences), and MCI gel (CHP 20P, 75–150 μm, Mitsubishi Chemical Co., Ltd., Tokyo, Japan) were used for column chromatography (CC). Reversed-phase (RP) HPLC was performed on an Agilent 1260 series liquid chromatography equipped with a DAD G1315D detector and an Agilent Eclipse XDB-C_18_ column (5 μm, 9.4 × 250 mm). In contrast, chiral HPLC separation was operated on the chromatography equipped with CHIRALPAK IB N-3 column (5 μm, 4.6 × 250 mm). All solvents used for CC and HPLC were analytical grade (Shanghai Chemical Reagents Co., Ltd., Shanghai, China) and chromatographic grade (DiKMA Technologies Inc., CA, USA), respectively.

**Animal materials**. See ref [11].

**Extraction and isolation**. For the extraction and preliminary fractionation of the extract, see ref [11]. The extract was separated by MCI column chromatography (CC) eluted with a MeOH/H_2_O gradient solvent system (10% to 100%) to yield six fractions (A–F). The fraction B (570 mg) was then fractionated into subfractions B1–B6 by Sephadex LH-20 CC eluted with CH_2_Cl_2_. The subfraction B4 (96.2 mg) was further separated by Sephadex LH-20 CC (petroleum ether (PE)/CH_2_Cl_2_/MeOH 2:1:1) to give subfractions B4A and B4B. The subfraction B4B (81.0 mg) was chromatographed by RP-HPLC (35% to 50% MeCN in H_2_O, 3.0 mL/min), yielding compounds **5** (4.6 mg, *t*_R_ = 9.9 min), **7** (0.5 mg, *t*_R_ = 13.8 min), (+)-**1** (1.2 mg, *t*_R_ = 18.9 min) and **2** (4.1 mg, *t*_R_ = 10.3 min). The subfraction B5 (80.1 mg) was purified by RP-HPLC (65% MeOH in H_2_O, 3.0 mL/min) to give **4** (2.7 mg, *t*_R_ = 16.2 min). The subfraction C2 (66.5 mg) was successively separated by silica gel CC (PE/Et_2_O 5:1 to 1:1) and RP-HPLC (75% MeCN in H_2_O, 3.0 mL/min) to give **3** (3.6 mg, *t*_R_ = 9.8 min). The subfraction E (34.9 mg) was successively separated by Sephadex LH-20 CC (PE/CH_2_Cl_2_/MeOH, 2:1:1) and RP-HPLC (55% MeCN in H_2_O, 3.0 mL/min) to afford compound **6** (1.3 mg, *t*_R_ = 12.4 min).

The separation of racemic mixtures (**4** and **5**) was performed on chiral HPLC equipped with a CHIRALPAK IB N-3 column eluted with MeCN/H_2_O [**4**: 25:75, **5**: 15:85, respectively, 1.0 mL/min] at room temperature, yielding (+)-**4** (0.8 mg, *t*_R_ = 18.4 min) and (−)-**4** (0.8 mg, *t*_R_ = 16.7 min), (+)-**5** (0.8 mg, *t*_R_ = 30.4 min), (−)-**5** (0.7 mg, *t*_R_ = 31.9 min), respectively.

(+)-Ximaocavernosin P [(+)-**1**]: colorless oil; 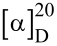 +169.6 (*c* 0.12, CHCl_3_); UV (MeCN) λ_max_, nm (log ε): 245 (3.8) nm; ECD (MeCN) λ _max_, nm (Δε): 222 (−9.7), 257 (+7.2), 340 (+1.3); IR (KBr) ν_max_: 3435, 2956, 2927, 2872, 1644, 1383, 1261, 1109, 1037 cm^−1^; ^1^H and ^13^C NMR data, see Table 1; HRMS–ESI (*m/z*): [M + H]^+^ calcd for C_15_H_23_O_2_, 235.1693; found, 235.1700.

*ent*-4β,10α-Dihydroxyaromadendrane (**2**): colorless crystal; mp 117.0–118.0 °C; 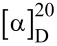 +23.8 (*c* 0.40, MeOH).

(+)-Maninsigin D [(+)-**4**]: white powder; 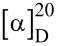 +26.0 (*c* 0.08, MeOH); ECD (MeCN) λ_max_, nm (Δε): 203 (−0.5), 218 (+0.2); IR (KBr) ν_max_: 2925, 1699, 1632, 1435, 1384, 1259, 1243, 1116, 1069 cm^−1^; ^1^H and ^13^C NMR data, see Table 1; HRMS–ESI (*m/z*): [M + H]^+^ calcd for C_15_H_23_O_2_, 235.1693; found, 235.1689.

(+)-Ximaocavernosin Q [(+)-**5**]: white powder; 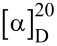 +8.8 (*c* 0.08, MeOH); ECD (MeCN) λ _max_, nm (Δε): 207 (−0.3), 216 (+0.1); IR (KBr) ν_max_: 3386, 2961, 2925, 2874, 1463, 1383, 1130, 993, 939, 820 cm^−1^; ^1^H and ^13^C NMR data, see Table 1; HRMS–EI (*m/z*): M^+^ calcd for C_15_H_22_O_2_, 234.1614; found, 234.1618.

(−)-Ximaocavernosin Q [(−)-**5**]: white powder; 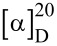 −8.3 (*c* 0.07, MeOH); ECD (MeCN) λ _max_, nm (Δε) 208 (+0.2); IR (KBr) ν_max_: 3386, 2961, 2925, 2874, 1463, 1383, 1130, 993, 939, 820 cm^−1^; ^1^H and ^13^C NMR data, see Table 1; HRMS–EI (*m/z*): M^+^ calcd for C_15_H_22_O_2_, 234.1614; found, 234.1618.

**X-ray crystallographic analysis**. Compound **2** was crystallized from MeCN at 4 °C. The crystallographic data for compound **2** was collected on a Bruker D8 Venture diffractometer using Cu Kα radiation (λ = 1.54178 Å). The collected data integration and reduction were processed with SAINT V8.37A software, and multi-scan absorption correction was performed using the SADABS program. The structure was solved using ShelXTL and refined on *F^2^* by the full-matrix least-squares technique using the SHELXL-2015 program package. The crystallographic data has been uploaded to the Cambridge Crystallographic Data Centre with CCDC number 2173439 (**2**). The data could be obtained free of charge via http://www.ccdc.cam.ac.uk/data_request/cif. Details of the crystallographic data were shown in Supporting Information File 1.

**Computational section**. TDDFT-ECD calculations. Conformational searches were conducted using the torsional sampling (MCMM) method and the OPLS_2005 force field. Conformers above 1% population were reoptimized at the B3LYP/6-311G** level of theory with IEFPCM (Polarizable Continuum Model using the Integral Equation Formalism variant) solvent model for acetonitrile. For the resulting geometries, ECD spectra were obtained by TDDFT calculations performed with the B3LYP/6-311G** level of theory with IEFPCM solvent model for acetonitrile. Finally, the Boltzmann-averaged ECD spectra were obtained with SpecDis1.62 [41].

**In vitro anti-inflammatory assay**. RAW264.7 cells, a murine macrophage cell line, was purchased from American Type Culture Collection (ATCC, Manassas, VA, USA). RAW264.7 cells were cultured in DMEM (Dulbecco’s modified Eagle medium) supplemented with 10% fetal bovine serum, antibiotics (100 U/mL penicillin and 100 U/mL streptomycin), and maintained at 37 °C in a humidified incubator of 5% CO_2_. RAW264.7 cells (1.6 × 10^5^/well) were seeded in a 24-well plate overnight for cell adherence. Cells were treated with LPS alone (100 ng/mL, dissolved in DMSO) or with compounds at the indicated concentrations for 24 h. Total RNA was harvested from cells using TRIzol^TM^ reagent (Invitrogen, USA) after being washed thrice with cold PBS. The total RNA (1 μg) was transcribed into cDNA in 20 μL reactions using the PrimeScript RT reagent kit (ABclonal, China) and then diluted to 200 μL. Real-time quantitative PCR (qPCR) was performed using SYBR GREEN QPCR KIT (Bimake, B21202) on Agilent Mx3000P following the manufacturer’s instructions. β-ACTIN was used as the normalization control. All reactions were performed in triplicate. The NF-κB inhibitor Bay 11-7082 (5 μM) was used as a positive control.

## Supporting Information

File 1 HPLC chromatograms of **4** and **5**, chiral separation of **4** and **5**, X-ray crystallographic data for **2**, spectra of compounds (+)-**1**, **4** and **5**, TDDFT-ECD calculation of compound (+)-**1**.
