# Supplementary material for: Anti-inflammatory aromadendrane- and cadinane-type sesquiterpenoids from the South China Sea sponge Acanthella cavernosa
Source: Beilstein J Org Chem. 2022 Jul 25;18:916–25. doi: 10.3762/bjoc.18.91 (PMC9344550; doi:10.3762/bjoc.18.91)
Supplement: File 1 — HPLC chromatograms of 4 and 5, chiral separation of 4 and 5, X-ray crystallographic data for 2, spectra of compounds (+)-1, 4 and 5, TDDFT-ECD calculation of compound (+)-1. [file Beilstein_J_Org_Chem-18-916-s001.pdf]

BEILSTEIN JOURNAL OF ORGANIC CHEMISTRY

Supporting Information

for

Anti-inflammatory aromadendrane- and cadinane-type sesquiterpenoids from the South China Sea sponge *Acanthella cavernosa*

Shou-Mao Shen, Qing Yang, Yi Zang, Jia Li, Xueting Liu and Yue-Wei Guo

Beilstein J. Org. Chem. **2022,** *18,* 916–925. doi:10.3762/bjoc.18.91

HPLC chromatograms of 4 and 5, chiral separation of 4 and 5, X-ray crystallographic data for 2, spectra of compounds (+)-1, 4 and 5, TDDFT-ECD calculation of compound (+)-1

License and Terms: This is a supporting information file under the terms of the Creative Commons Attribution License (https://creativecommons.org/licenses/by/4.0). Please note that the reuse, redistribution and reproduction in particular requires that the author(s) and source are credited and that individual graphics may be subject to special legal provisions.
The license is subject to the *Beilstein Journal of Organic Chemistry* terms and conditions: (https://www.beilstein-journals.org/bjoc/terms)

Table of contents

1. HPLC chromatograms of compounds **4** and **5**. S2

2. Chiral separation of compounds **4** and **5**. S3

3. X-ray crystallographic data for **2**. S5

4. NMR, MS and IR spectra of compounds (+)-**1**, **4**, and **5**. S6

    4.1. Original spectra of (+)-**1**. S6

    4.2. Original spectra of **4**. S10

    4.3. Original spectra of **5**. S14

5. TDDFT-ECD calculation of compound (+)-**1**. S20

    5.1 Computational section. S20

    5.2 Computational data of (2*S*,4*S*,5*R*,6*S*,7*S*)-[(+)-**1**]. S21

1. HPLC chromatograms of compounds 4 and 5.

Chromatographic conditions: An Agilent Eclipse XDB-C_{18} column (5 μm, 9.4 × 250 mm), MeCN:H_2O (35:65), 3.0 mL/min, 210 nm, (±)-**4** (t_R = 17.6 min), (±)-**5** (t_R = 10.6 min).

A

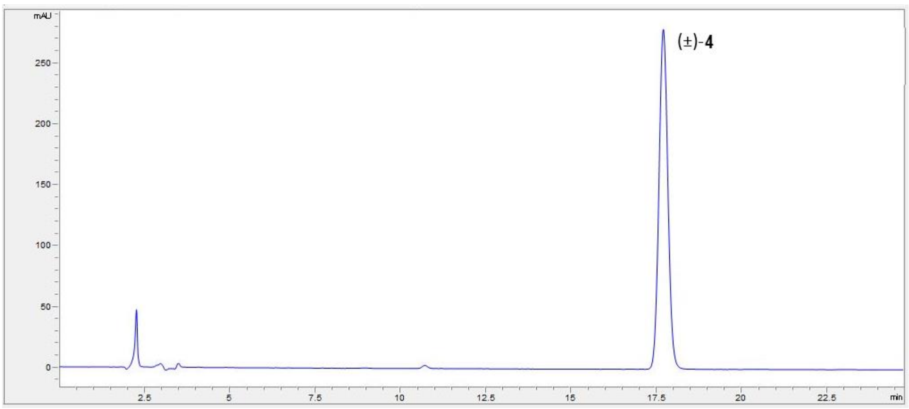

B

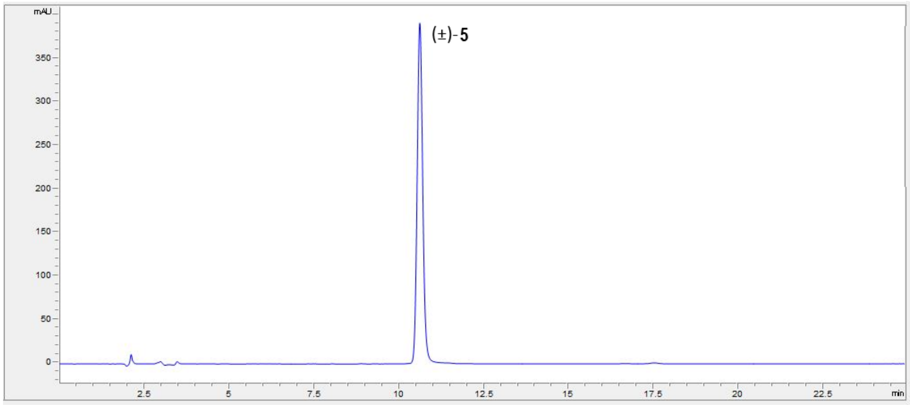

C

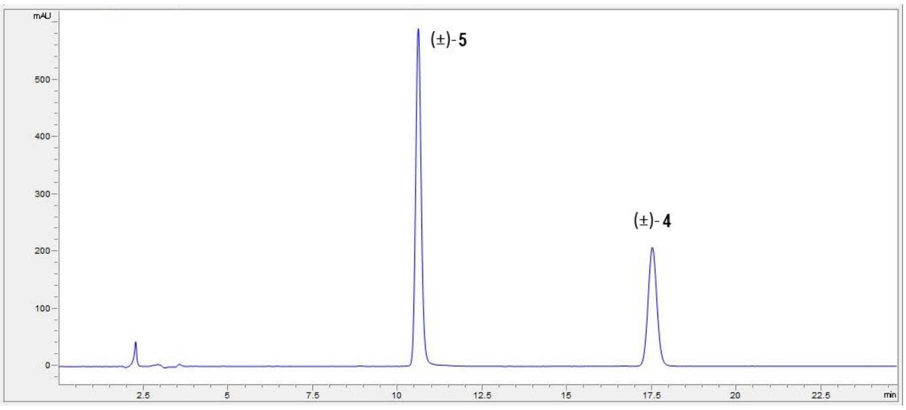

Figure S1. RP-HPLC chromatograms of compounds (±)-**4** (A), (±)-**5** (B) and mixture of (±)-**4** and (±)-**5** (C).

2. Chiral separation of compounds 4 and 5.

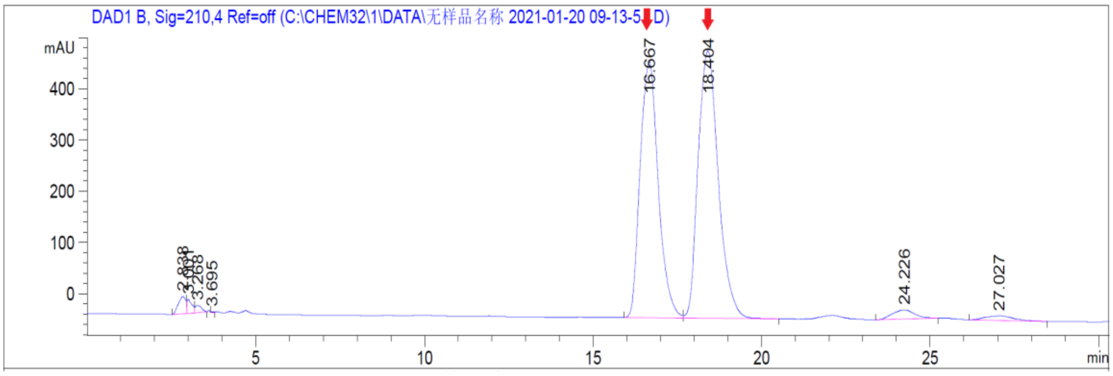

Figure S2. Chiral HPLC separation chromatography of compound **4**, (−)-**4**, t_R = 16.7 min and (+)-**4**, t_R = 18.4 min.

n	Average	Std.Dev.	Maximum	Minimum
6	26.042	0.4658	26.250	25.000

S.No	Sample ID	Time	Result	Scale	OR °Arc	WLG	Lg.mm	Conc.	Temp.	Comment
1	JD5D-2	08:25:52 PM	25.000	SR	0.020	589	100.00	0.080	20.0	
2	JD5D-2	08:26:04 PM	26.250	SR	0.021	589	100.00	0.080	20.0	
3	JD5D-2	08:26:12 PM	26.250	SR	0.021	589	100.00	0.080	20.0	
4	JD5D-2	08:26:20 PM	26.250	SR	0.021	589	100.00	0.080	19.9	
5	JD5D-2	08:26:27 PM	26.250	SR	0.021	589	100.00	0.080	19.9	
6	JD5D-2	08:26:35 PM	26.250	SR	0.021	589	100.00	0.080	19.9	

Figure S3. Specific optical rotation of (+)-**4**.

n	Average	Std.Dev.	Maximum	Minimum
6	-24.167	0.5893	-23.750	-25.000

S.No	Sample ID	Time	Result	Scale	OR °Arc	WLG	Lg.mm	Conc.	Temp.	Comment
1	JD5D-1	08:53:26 PM	-23.750	SR	-0.019	589	100.00	0.080	19.8	
2	JD5D-1	08:53:34 PM	-23.750	SR	-0.019	589	100.00	0.080	19.8	
3	JD5D-1	08:53:42 PM	-23.750	SR	-0.019	589	100.00	0.080	19.8	
4	JD5D-1	08:53:49 PM	-23.750	SR	-0.019	589	100.00	0.080	19.8	
5	JD5D-1	08:53:57 PM	-25.000	SR	-0.020	589	100.00	0.080	19.8	
6	JD5D-1	08:54:05 PM	-25.000	SR	-0.020	589	100.00	0.080	19.8	

Figure S4. Specific optical rotation of (−)-**4**.

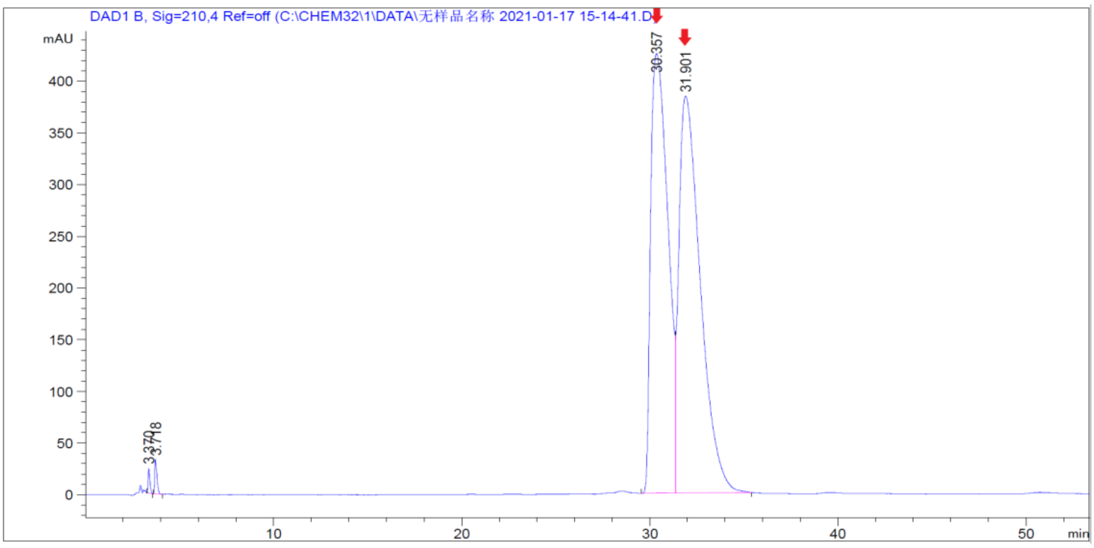

Figure S5. Chiral HPLC separation chromatography of compound **5**, (+)-**5**, t_R = 30.4 min, (−)-**5**, t_R = 31.9 min.

n	Average	Std.Dev.	Maximum	Minimum
6	8.750	0.0000	8.750	8.750

S.No	Sample ID	Time	Result	Scale	OR ° Arc	WLG	Lg.mm	Conc.	Temp.	Comment
1	JD4B5A1	06:56:24 PM	8.750	SR	0.007	589	100.00	0.080	20.2	
2	JD4B5A1	06:56:31 PM	8.750	SR	0.007	589	100.00	0.080	20.1	
3	JD4B5A1	06:56:38 PM	8.750	SR	0.007	589	100.00	0.080	20.1	
4	JD4B5A1	06:56:45 PM	8.750	SR	0.007	589	100.00	0.080	20.1	
5	JD4B5A1	06:56:51 PM	8.750	SR	0.007	589	100.00	0.080	20.0	
6	JD4B5A1	06:56:58 PM	8.750	SR	0.007	589	100.00	0.080	20.0	

Figure S6. Specific optical rotation of (+)-**5**.

n	Average	Std.Dev.	Maximum	Minimum
6	-8.333	0.5322	-7.143	-8.571

S.No	Sample ID	Time	Result	Scale	OR ° Arc	WLG	Lg.mm	Conc.	Temp.	Comment
1	JD4B5A2	07:16:56 PM	-8.571	SR	-0.006	589	100.00	0.070	19.8	
2	JD4B5A2	07:17:03 PM	-8.571	SR	-0.006	589	100.00	0.070	19.8	
3	JD4B5A2	07:17:10 PM	-8.571	SR	-0.006	589	100.00	0.070	19.8	
4	JD4B5A2	07:17:23 PM	-8.571	SR	-0.006	589	100.00	0.070	19.8	
5	JD4B5A2	07:17:29 PM	-8.571	SR	-0.006	589	100.00	0.070	19.8	
6	JD4B5A2	07:17:36 PM	-7.143	SR	-0.005	589	100.00	0.070	19.8	

Figure S7. Specific optical rotation of (−)-**5**.

3. X-ray crystallographic data for 2.

The crystal of **2** was recrystallized from MeCN at 4 °C. X-ray analysis was carried out on a Bruker D8 Venture diffractometer with Cu Kα radiation (λ = 1.54178 Å) at 170 K. $C_{15}H_{26}O_2$, M_r = 238.36, orthorhombic, crystal size 0.15 × 0.08 × 0.05 mm^3, space group $C222_1$, a = 10.6819(3) Å, b = 16.0392(3) Å, c = 16.6572(4) Å, V = 2853.86(12) Å^3, Z = 8, ρ_{calcd} = 1.110 g/cm^3, F(000) = 1056.0, 15570 collected reflections, 2865 independent reflections (R_{int} = 0.0397, R_{sigma} = 0.0243), final R indexes [I ≥ 2σ (I)]: R_1 = 0.0501, wR_2 = 0.1269), final R indexes (all data): R_1 = 0.0557, wR_2 = 0.1329, Flack parameter = 0.00(11). Crystallographic data (excluding structure factors) for the structure in this paper have been deposited with the Cambridge Crystallographic Data Center as supplementary publication CCDC 2173439.

4. NMR, MS and IR spectra of compounds (+)-1, 4, and 5.

4.1. Original spectra of (+)-**1**.

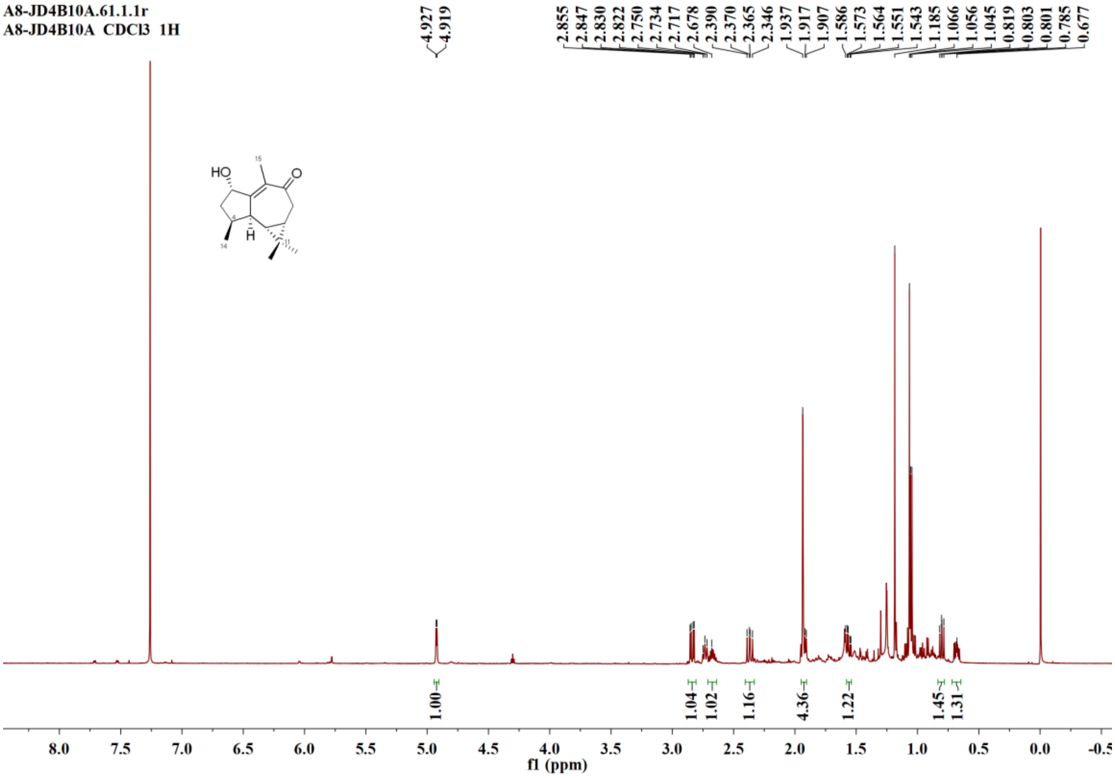

Figure S8. ^{1}H NMR spectrum (600 MHz) of (+)-**1** in $CDCl_3$.

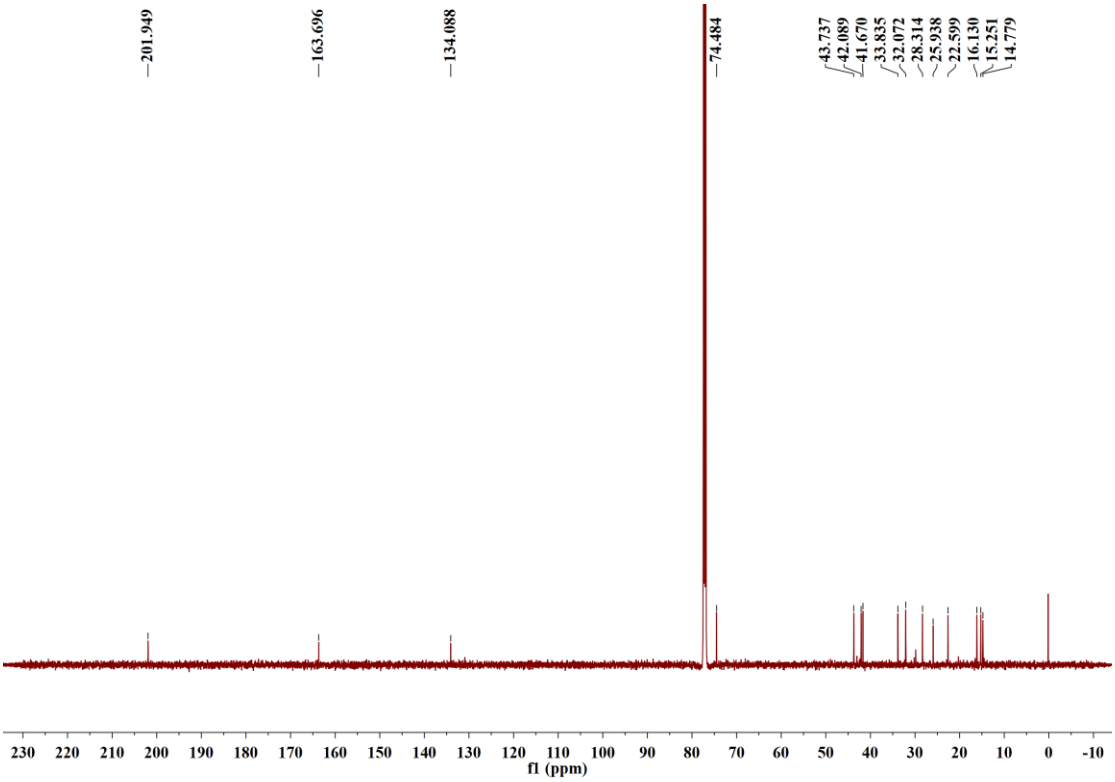

Figure S9. ^{13}C NMR spectrum (125 MHz) of (+)-**1** in $CDCl_3$.

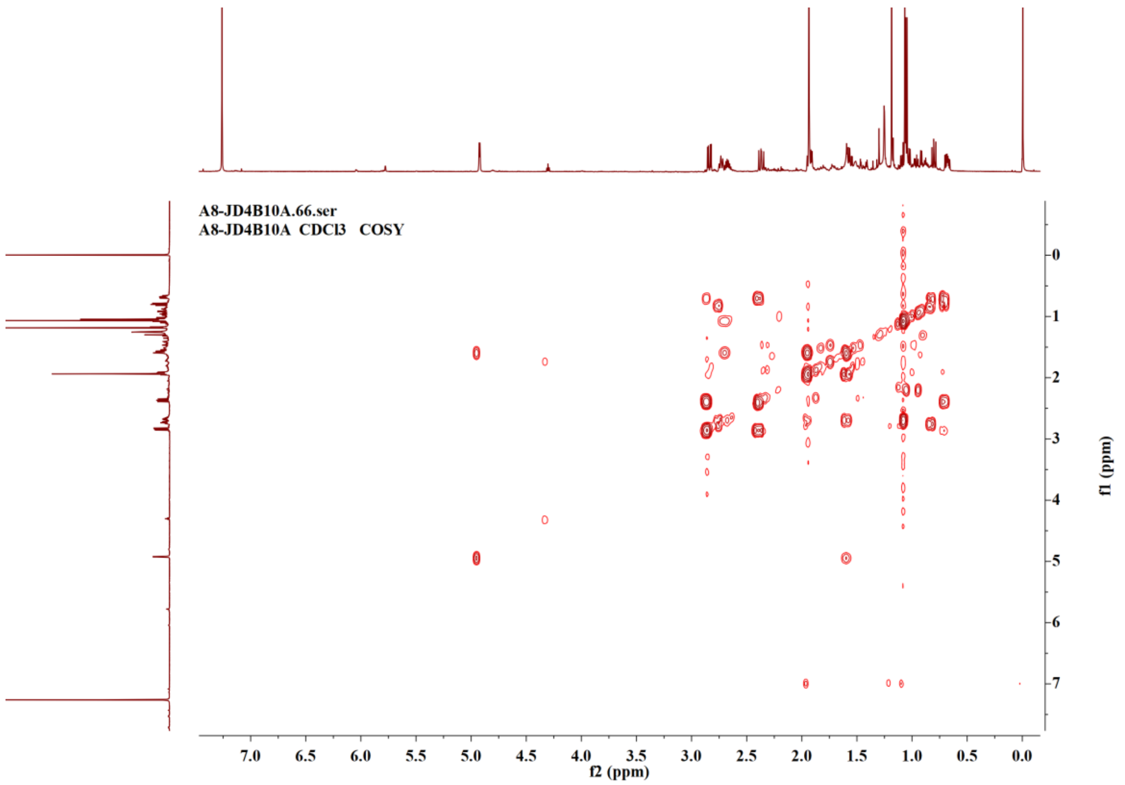

Figure S10. ^{1}H-^{1}H COSY spectrum of (+)-**1** in $CDCl_3$.

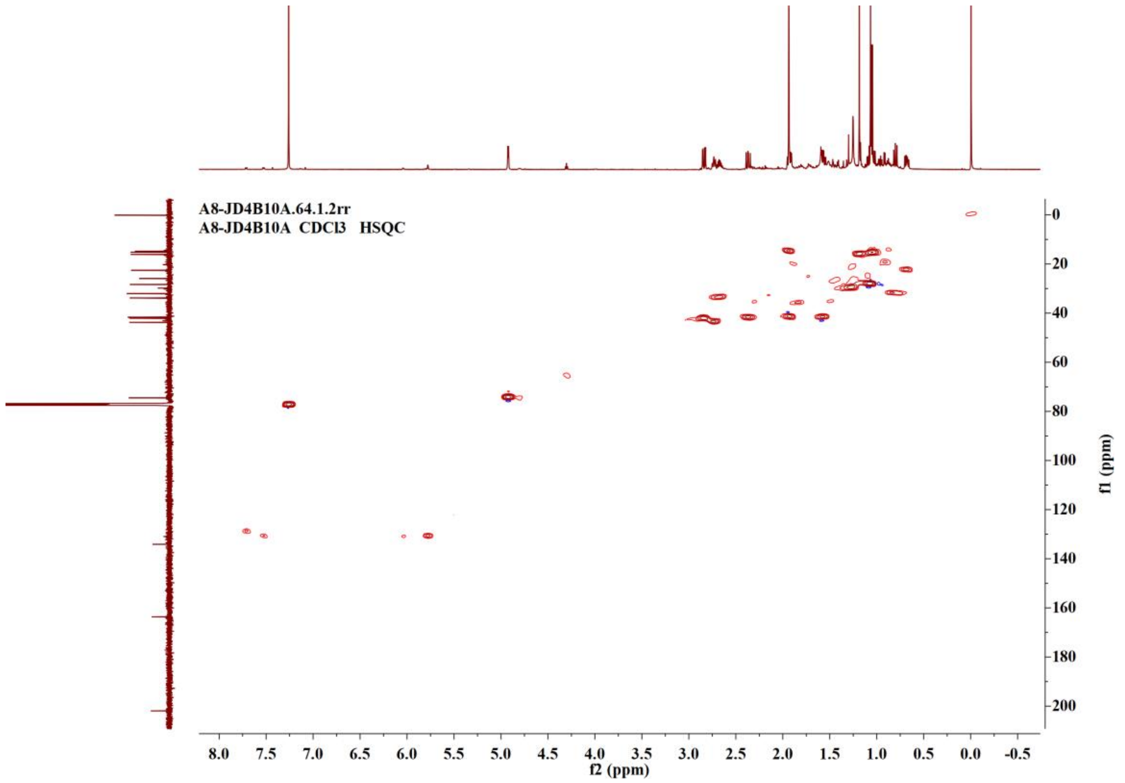

Figure S11. HSQC spectrum of (+)-**1** in $CDCl_3$.

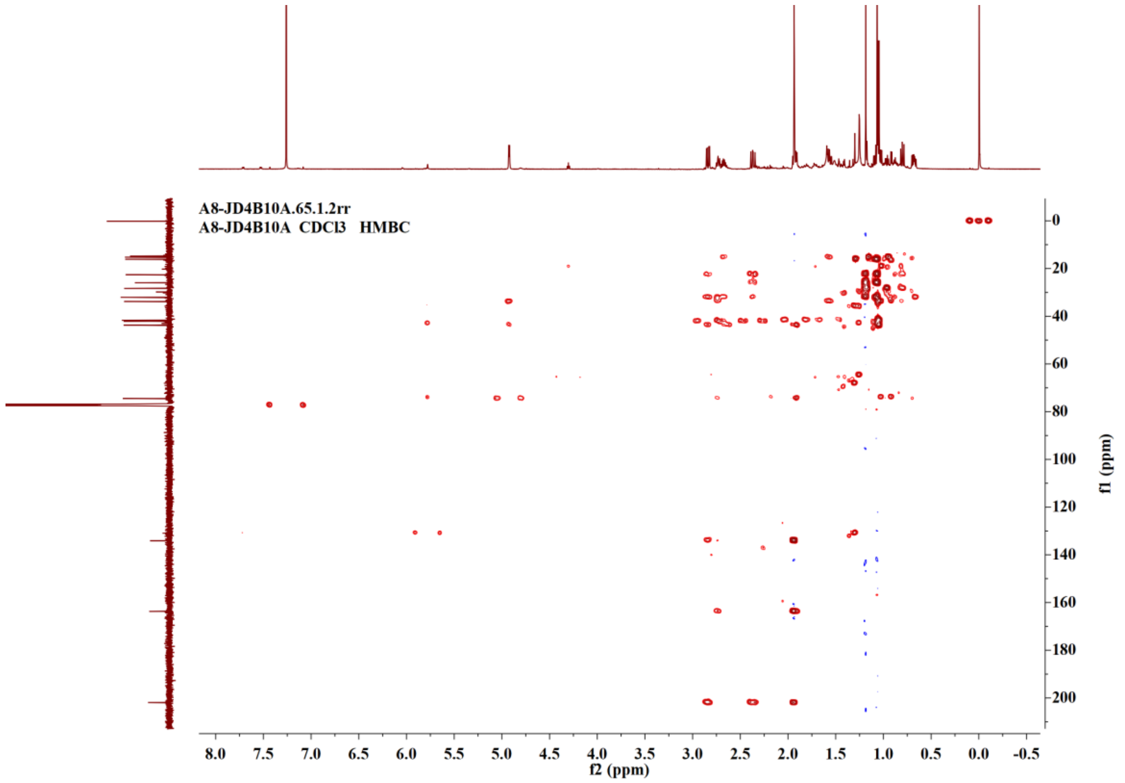

Figure S12. HMBC spectrum of (+)-**1** in $CDCl_3$.

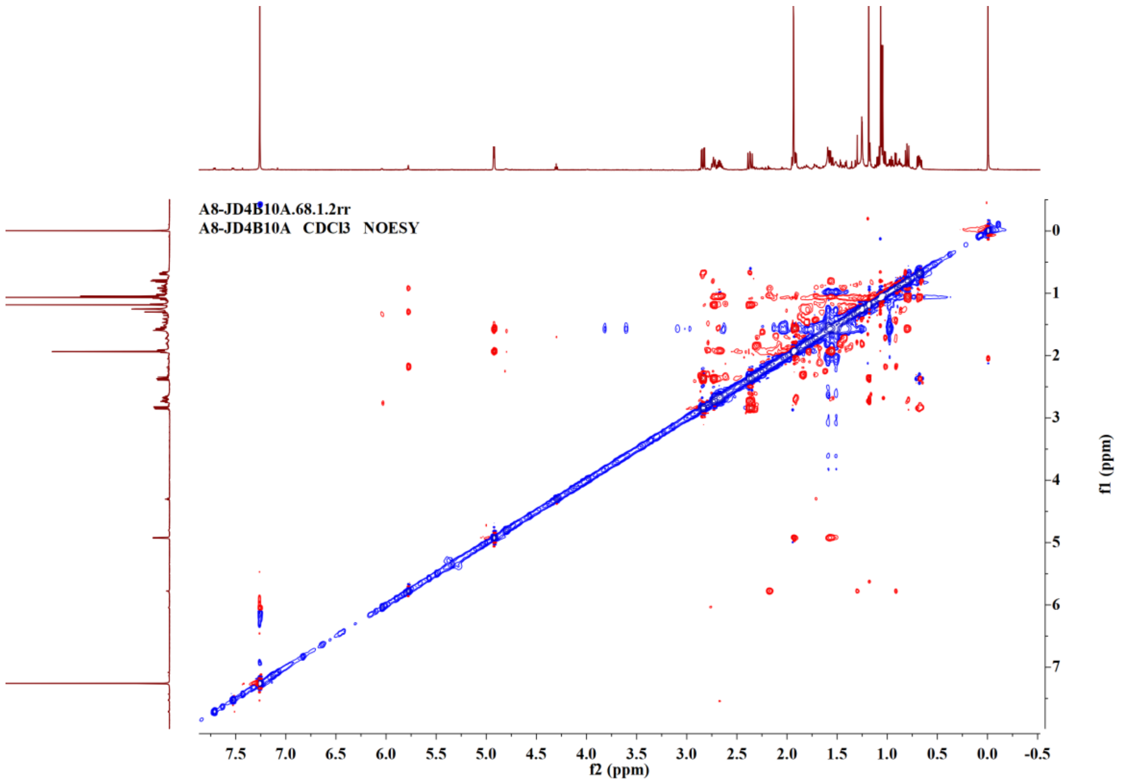

Figure S13. NOESY spectrum of (+)-**1** in $CDCl_3$.

Qualitative Analysis Report

**Data Filename**	ESIH202100485.d	**Sample Name**	A8-JD4B10A
**Sample ID**		**Position**	P1-A1
**Instrument Name**	Agilent G6520 Q-TOF	**Acq Method**	20160322_MS_ESIH_POS_1min.m
**Acquired Time**	1/22/2021 17:18:11	**IRM Calibration Status**	Success
**DA Method**	small molecular data analysis method.m	**Comment**	ESIH by ZZY

User Spectra

Fragmentor Voltage	Collision Energy	Ionization Mode
135	0	ESI

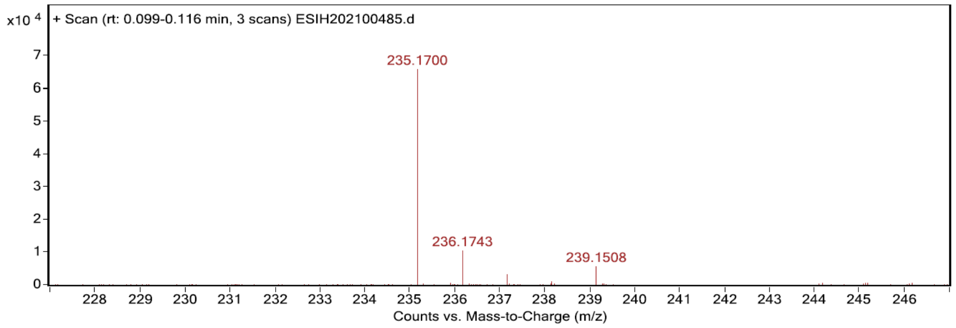

Formula Calculator Results

m/z	Calc m/z	Diff (mDa)	Diff (ppm)	Ion Formula	Ion
235.17	235.1693	-0.71	-3.03	C15 H23 O2	(M+H)+

--- End Of Report ---

Figure S14. HRESIMS of (+)-**1**.

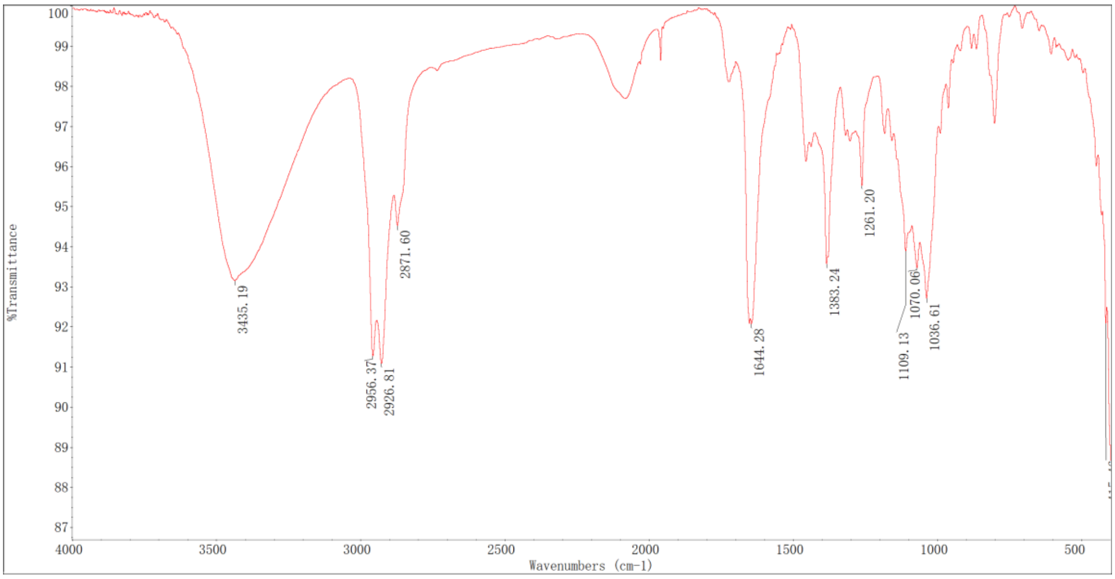

Figure S15. IR spectrum of (+)-**1**.

4.2. original spectra of **4**.

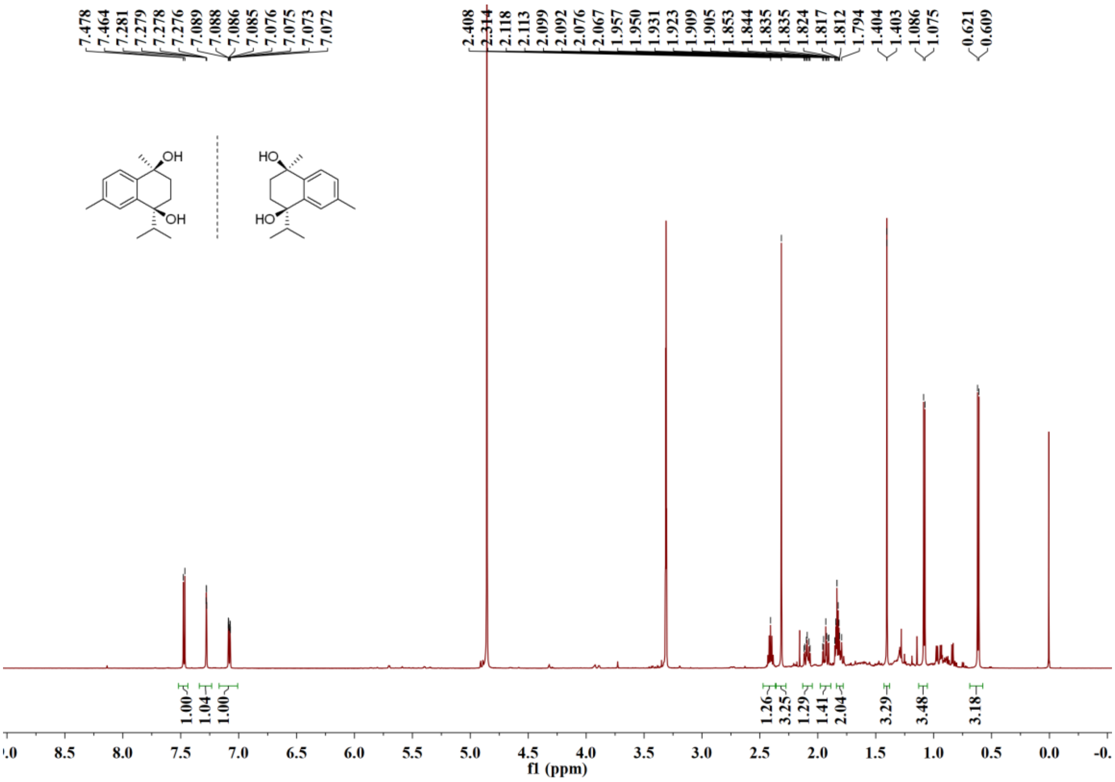

Figure S16. ^{1}H NMR spectrum (600 MHz) of **4** in CD_3OD.

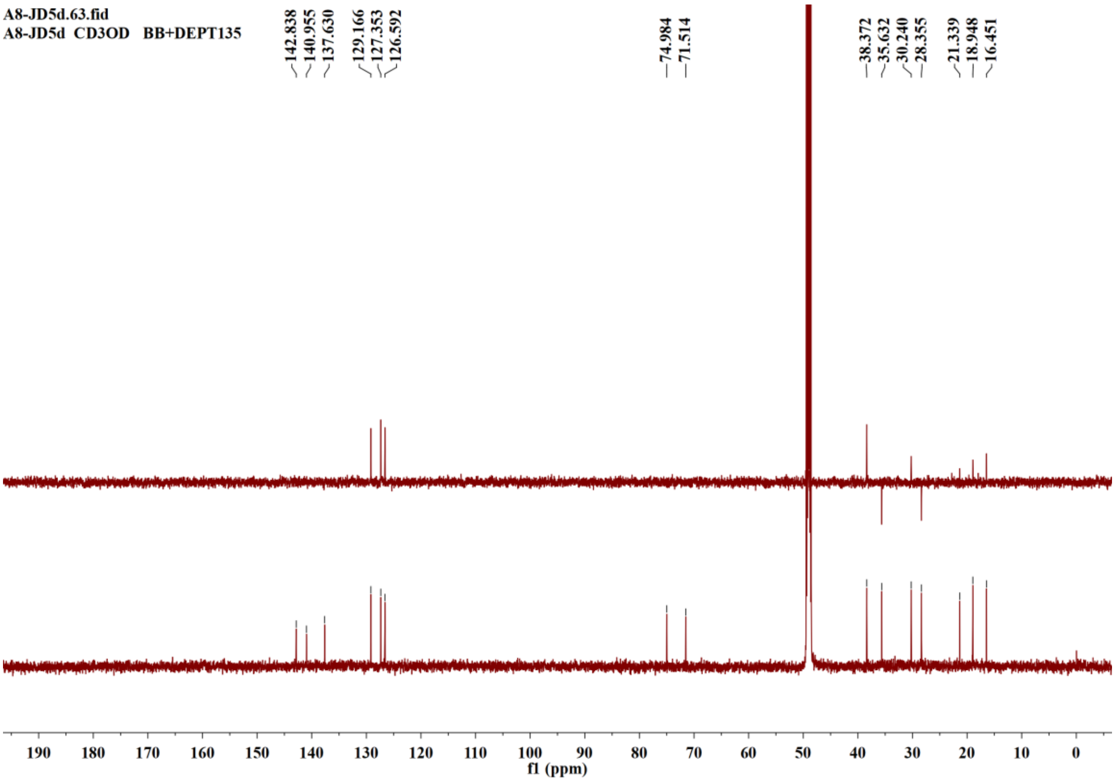

Figure S17. ^{13}C NMR spectrum (125 MHz) of **4** in CD_3OD.

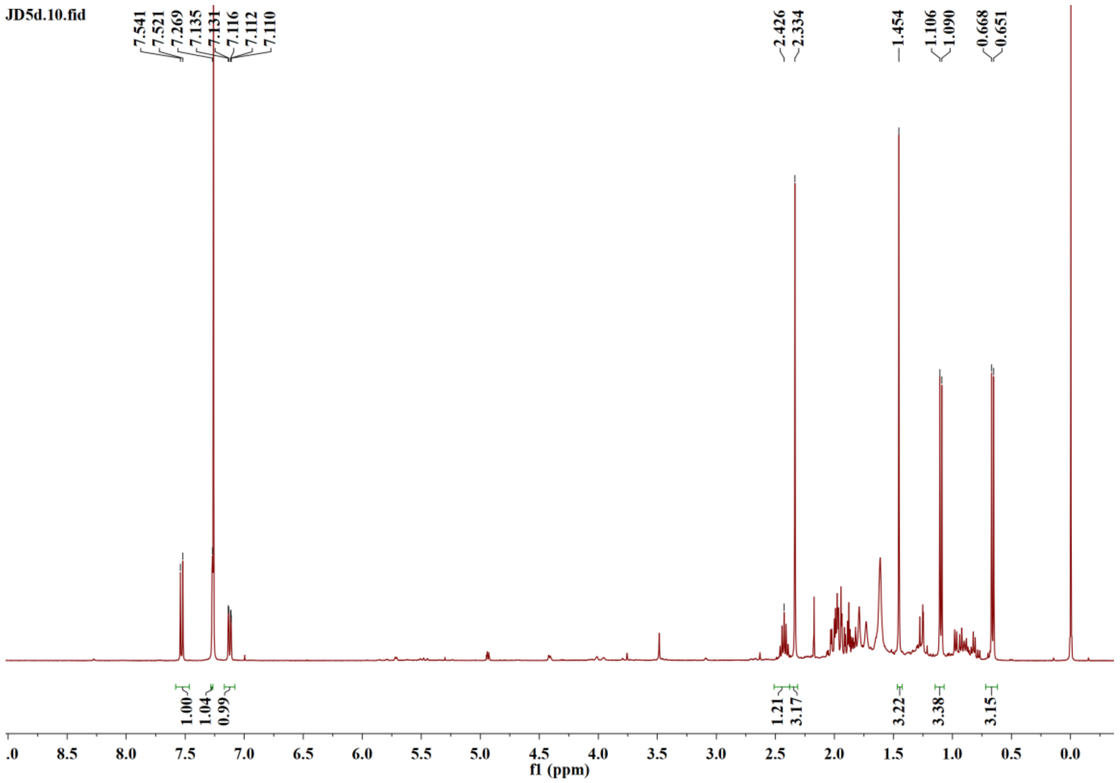

Figure S18. ^{1}H NMR spectrum (400 MHz) of **4** in $CDCl_3$.

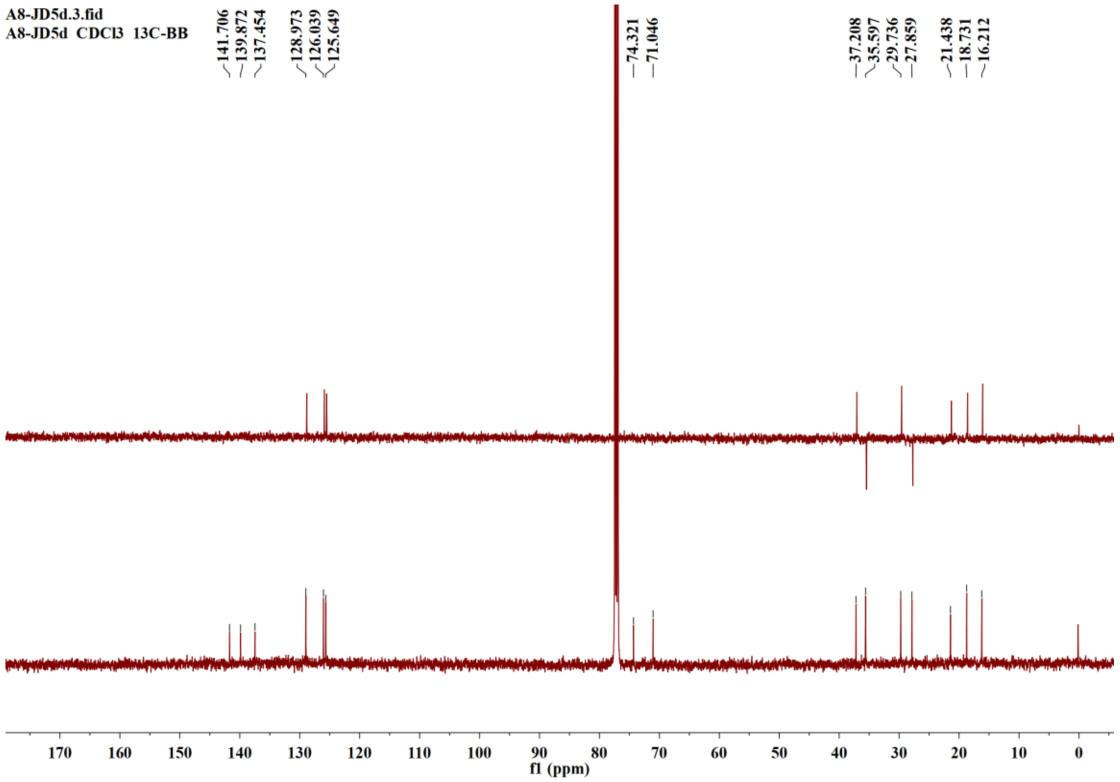

Figure S19. ^{13}C NMR spectrum (150 MHz) of **4** in $CDCl_3$.

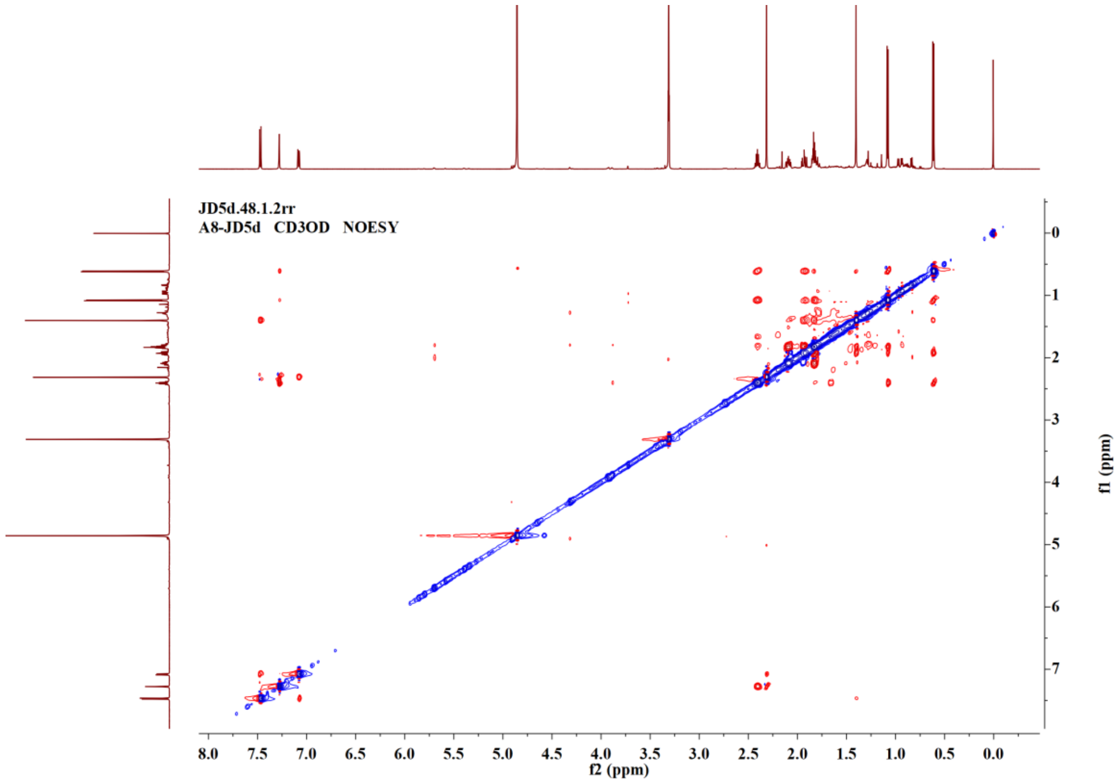

Figure S20. NOESY spectrum of **4** in CD_3OD.

Qualitative Analysis Report

**Data Filename**	ESIH202100175.d	**Sample Name**	A8-JD5Db
**Sample ID**		**Position**	P1-A3
**Instrument Name**	Agilent G6520 Q-TOF	**Acq Method**	20160322_MS_ESIH_POS_1min.m
**Acquired Time**	1/12/2021 19:13:02	**IRM Calibration Status**	Success
**DA Method**	small molecular data analysis method.m	**Comment**	ESIH by ZZY

User Spectra

Fragmentor Voltage	Collision Energy	Ionization Mode
135	0	ESI

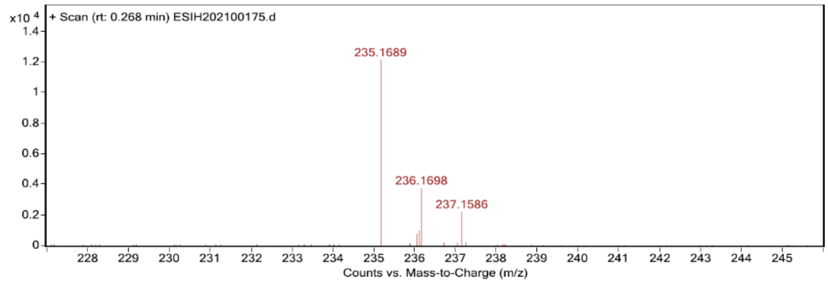

Formula Calculator Results

m/z	Calc m/z	Diff (mDa)	Diff (ppm)	Ion Formula	Ion
235.1689	235.1693	0.32	1.34	C15 H23 O2	(M+H)+

--- End Of Report ---

Figure S21. HRESIMS of **4**.

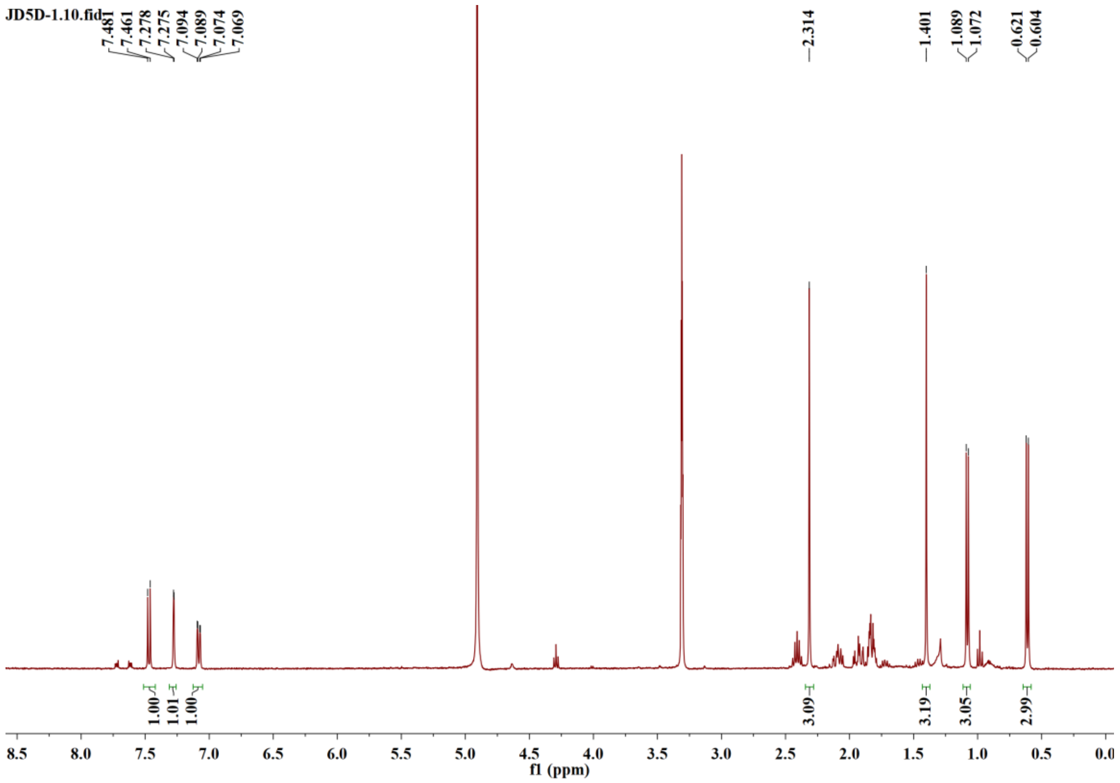

Figure S22. ^{1}H NMR spectrum (400 MHz) of (+)-**4** in CD_3OD.

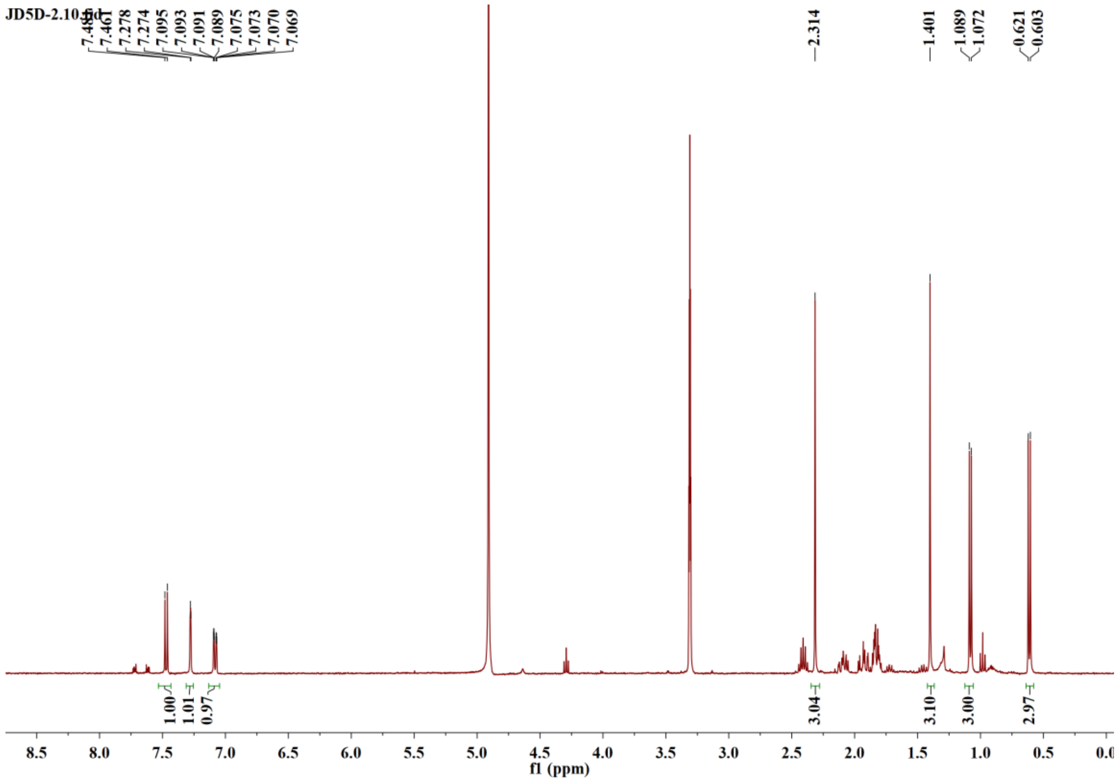

Figure S23. ^{1}H NMR spectrum (400 MHz) of (−)-**4** in CD_3OD.

4.3. original spectra of **5**.

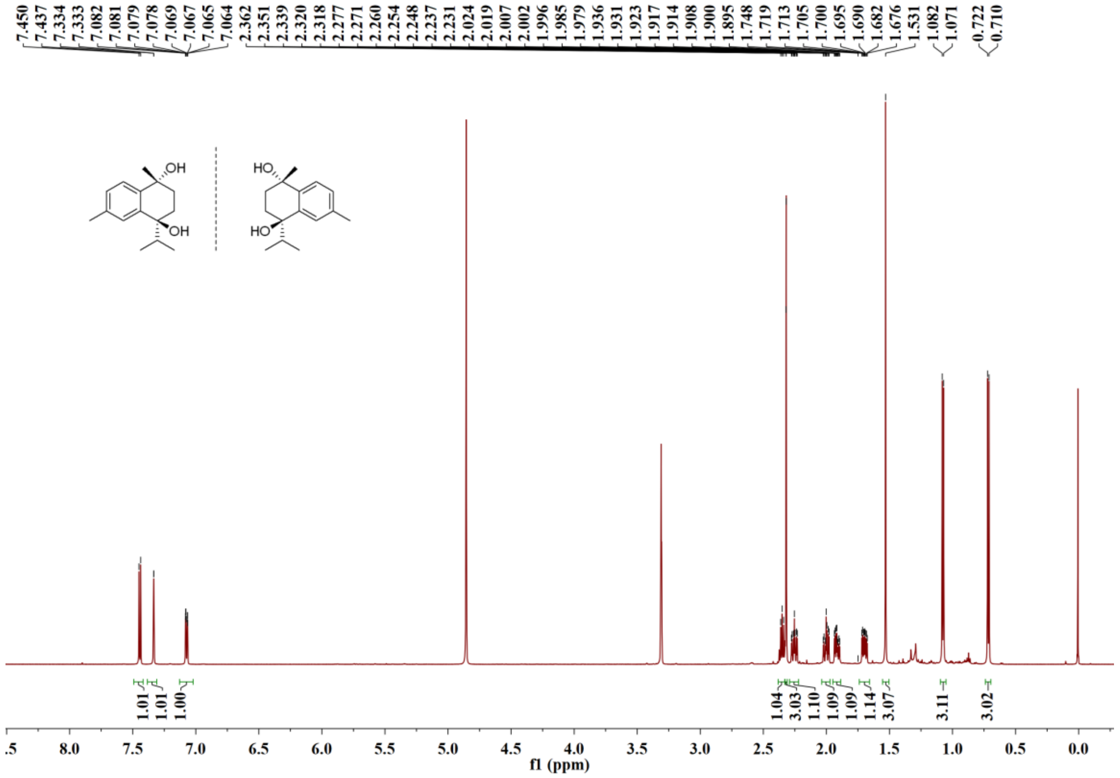

Figure S24. ^{1}H NMR spectrum (600 MHz) of **5** in CD_3OD.

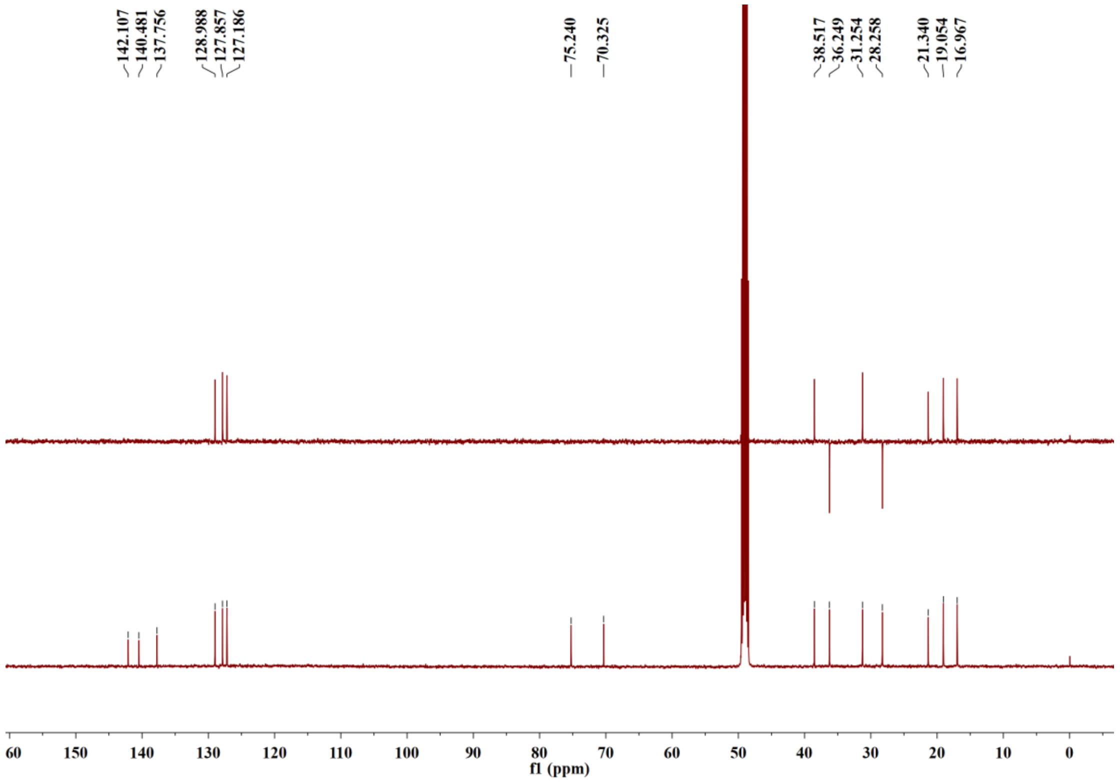

Figure S25. ^{13}C NMR spectrum (125 MHz) of **5** in CD_3OD.

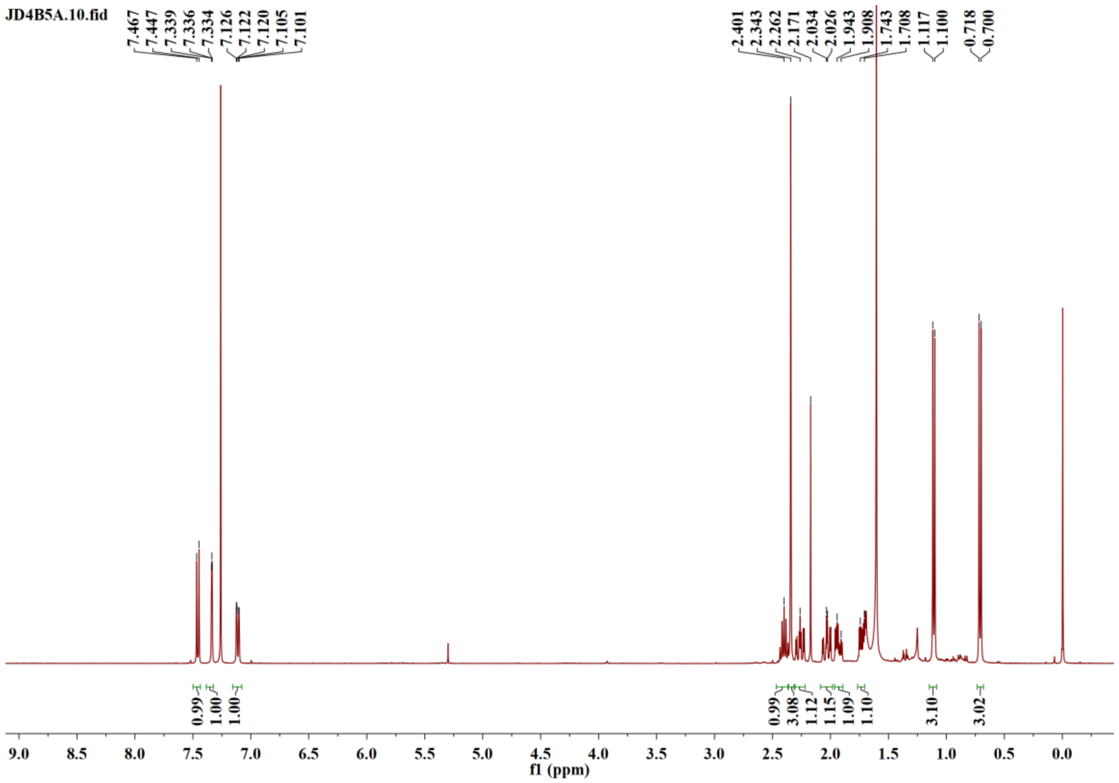

Figure S26. ^{1}H NMR spectrum (400 MHz) of **5** in $CDCl_3$.

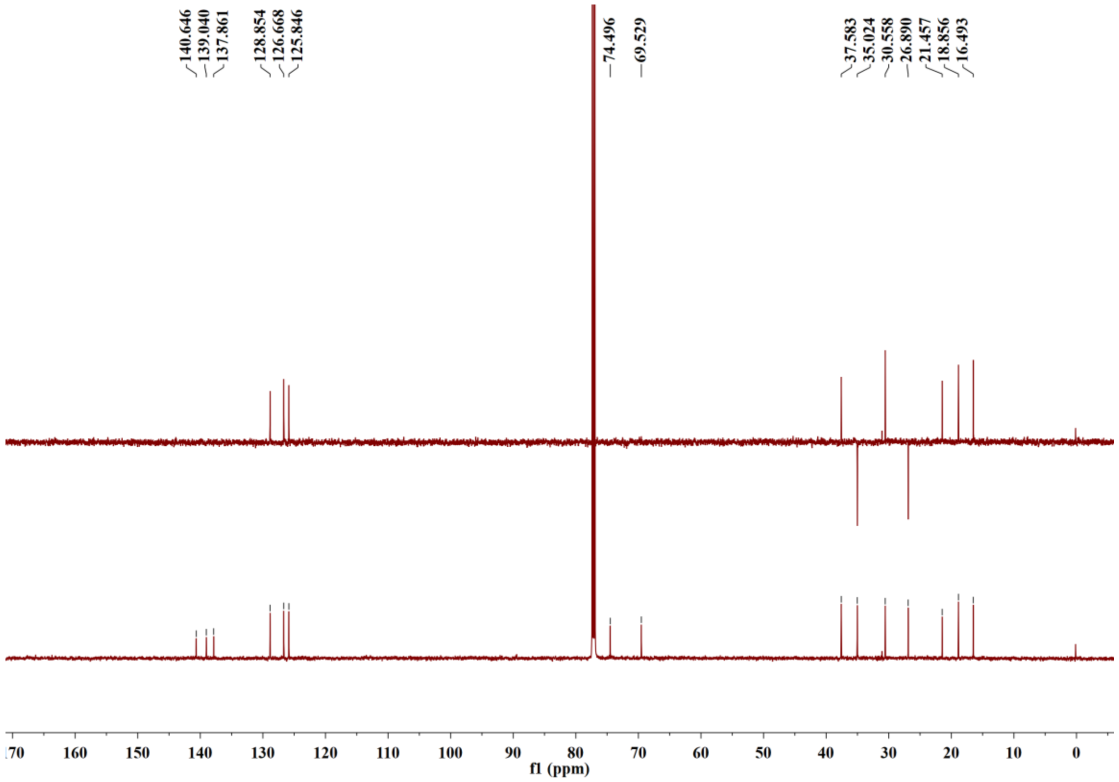

Figure S27. ^{13}C NMR spectrum (150 MHz) of **5** in $CDCl_3$.

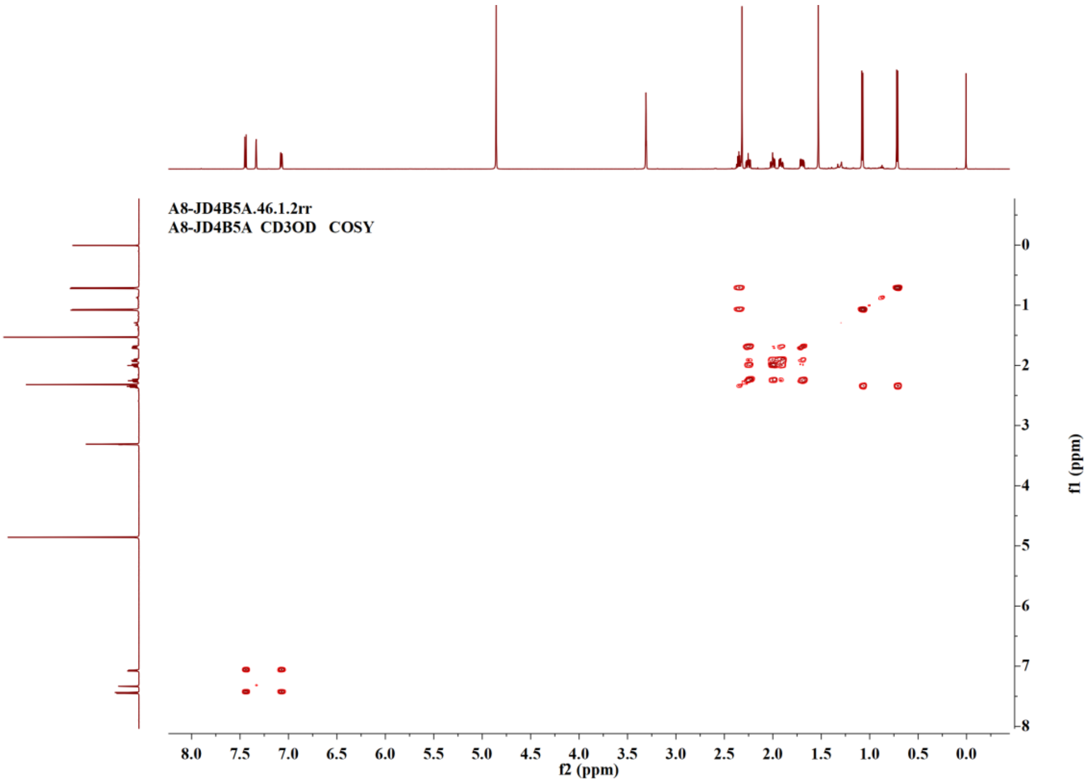

Figure S28. ^{1}H-^{1}H COSY spectrum of **5** in CD_3OD.

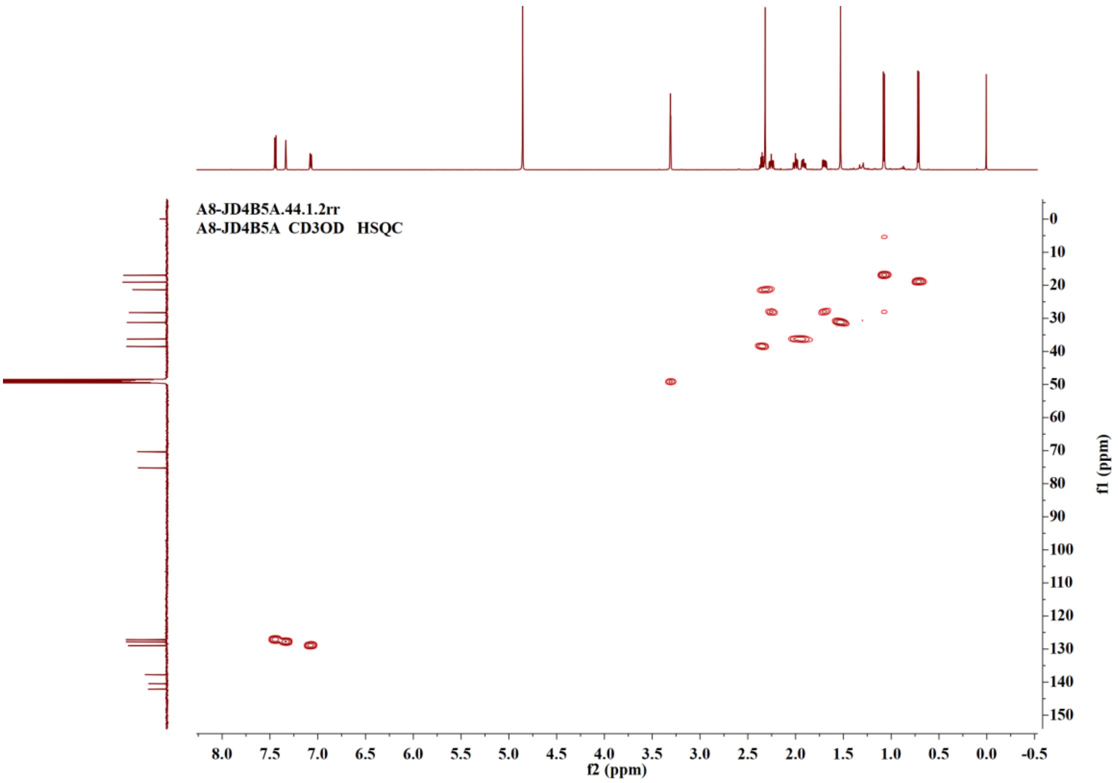

Figure S29. HSQC spectrum of **5** in CD_3OD.

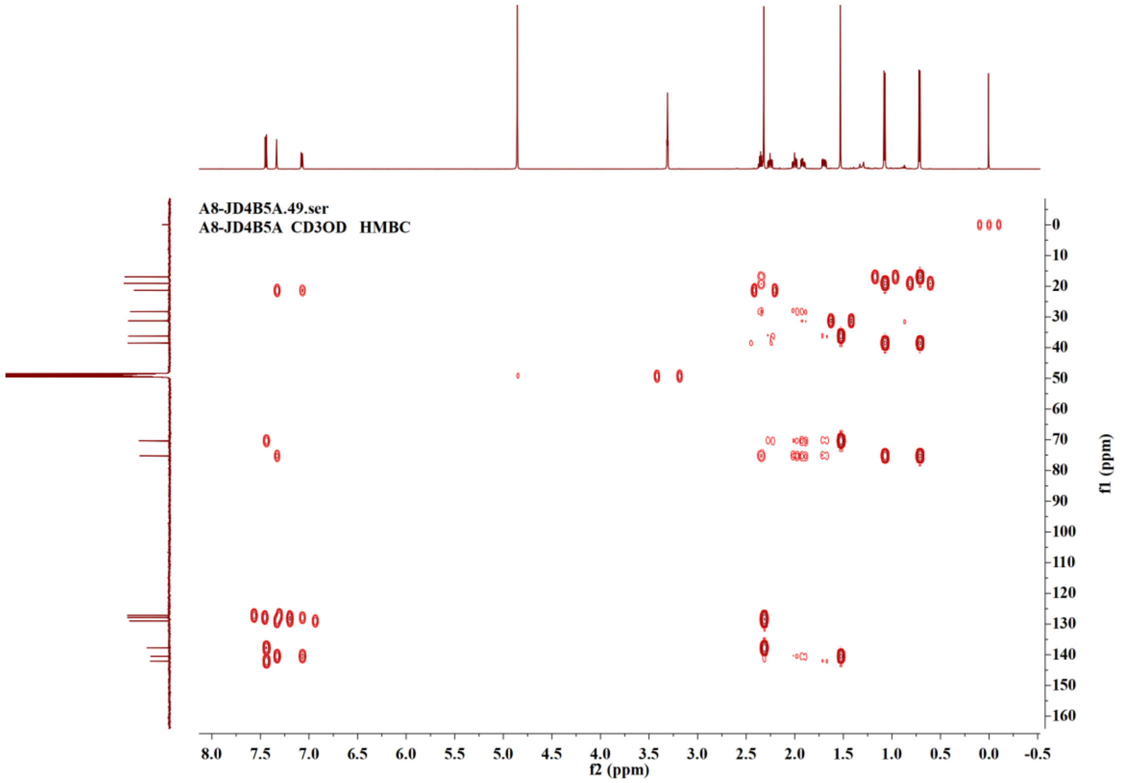

Figure S30. HMBC spectrum of **5** in CD_3OD.

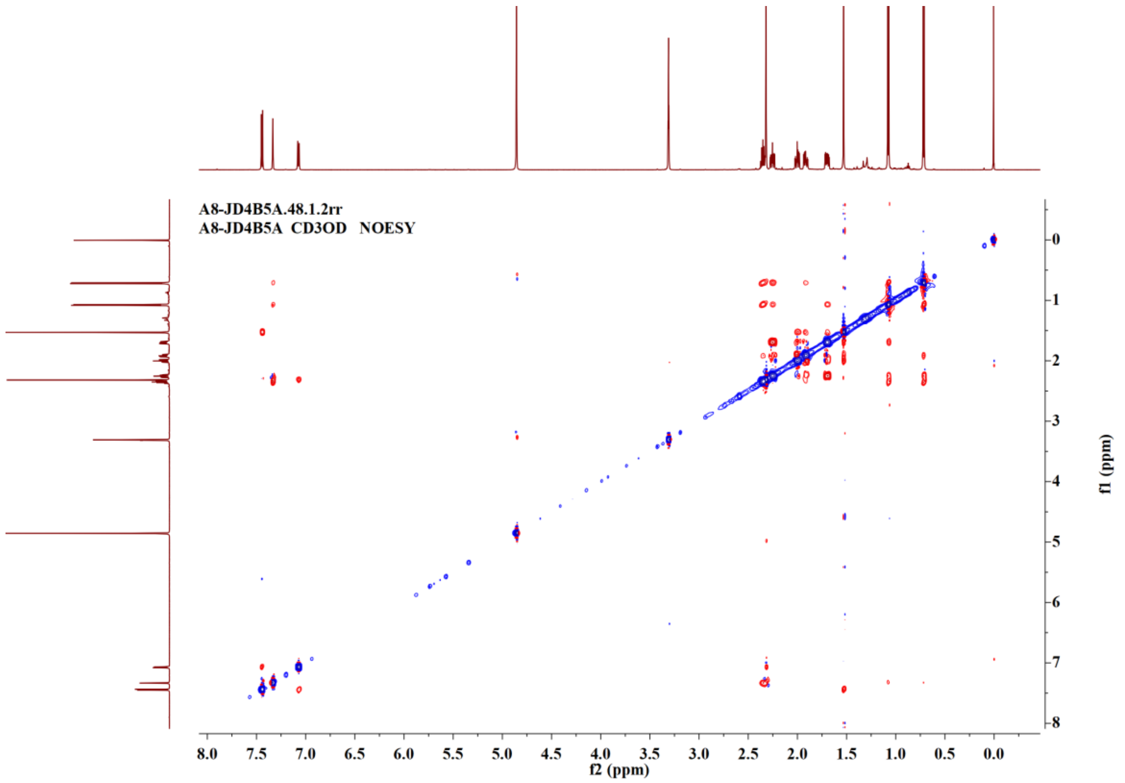

Figure S31. NOESY spectrum of **5** in CD_3OD.

EIH2020-12-25-05-GYW1 A8-JD4B5A -c1#4 RT: 0.62
T: + c EI Full ms [49.50-800.50]
m/z= 48-803

m/z	Intensity	Relative	Theo. Mass	Delta (mmu)	RDB equiv.	Composition
173.0963	1330209.0	100.00	173.0961	0.24	6.5	$C_{12}H_{13}O_1$
173.1332	11638.0	0.87	173.1325	0.72	5.5	$C_{13}H_{17}$
174.0995	183363.0	13.78	174.1039	-4.42	6.0	$C_{12}H_{14}O_1$
175.0749	36995.0	2.78	175.0754	-0.48	6.5	$C_{11}H_{11}O_2$
175.1078	25658.0	1.93	175.1117	-3.96	5.5	$C_{12}H_{15}O_1$
176.0819	18183.0	1.37	176.0832	-1.33	6.0	$C_{11}H_{12}O_2$
176.1184	7558.0	0.57	176.1196	-1.19	5.0	$C_{12}H_{16}O_1$
177.0906	19059.0	1.43	177.0910	-0.43	5.5	$C_{11}H_{13}O_2$
177.1279	10981.0	0.83	177.1274	0.51	4.5	$C_{12}H_{17}O_1$
178.0781	23030.0	1.73	178.0777	0.41	10.0	$C_{14}H_{10}$
179.0835	16512.0	1.24	179.0855	-1.98	9.5	$C_{14}H_{11}$
180.0927	8598.0	0.65	180.0934	-0.63	9.0	$C_{14}H_{12}$
181.1011	40637.0	3.05	181.1012	-0.08	8.5	$C_{14}H_{13}$
182.1087	15964.0	1.20	182.1090	-0.29	8.0	$C_{14}H_{14}$
183.0824	7804.0	0.59	183.0804	1.95	8.5	$C_{13}H_{11}O_1$
183.1174	504990.0	37.96	183.1168	0.56	7.5	$C_{14}H_{15}$
184.1204	85986.0	6.46	184.1247	-4.24	7.0	$C_{14}H_{16}$
185.0937	8215.0	0.62	185.0961	-2.35	7.5	$C_{13}H_{13}O_1$
187.0760	9611.0	0.72	187.0754	0.61	7.5	$C_{12}H_{11}O_2$
187.1100	12076.0	0.91	187.1117	-1.71	6.5	$C_{13}H_{15}O_1$
189.0910	17607.0	1.32	189.0910	-0.06	6.5	$C_{12}H_{13}O_2$
191.1060	185773.0	13.97	191.1067	-0.65	5.5	$C_{12}H_{15}O_2$
193.0963	7667.0	0.58	193.1012	-4.87	9.5	$C_{15}H_{13}$
194.1264	8215.0	0.62	194.1301	-3.71	4.0	$C_{12}H_{18}O_2$
196.1233	8516.0	0.64	196.1247	-1.31	8.0	$C_{15}H_{16}$
198.1397	208666.0	15.69	198.1403	-0.64	7.0	$C_{15}H_{18}$
199.1437	35982.0	2.70	199.1481	-4.42	6.5	$C_{15}H_{19}$
201.1280	35298.0	2.65	201.1274	0.59	6.5	$C_{14}H_{17}O_1$
202.1327	7968.0	0.60	202.1352	-2.51	6.0	$C_{14}H_{18}O_1$
216.1504	8406.0	0.63	216.1509	-0.42	6.0	$C_{15}H_{20}O_1$
234.1618	11227.0	0.84	234.1614	0.36	5.0	$C_{15}H_{22}O_2$

Figure S32. HRESIMS of **5**.

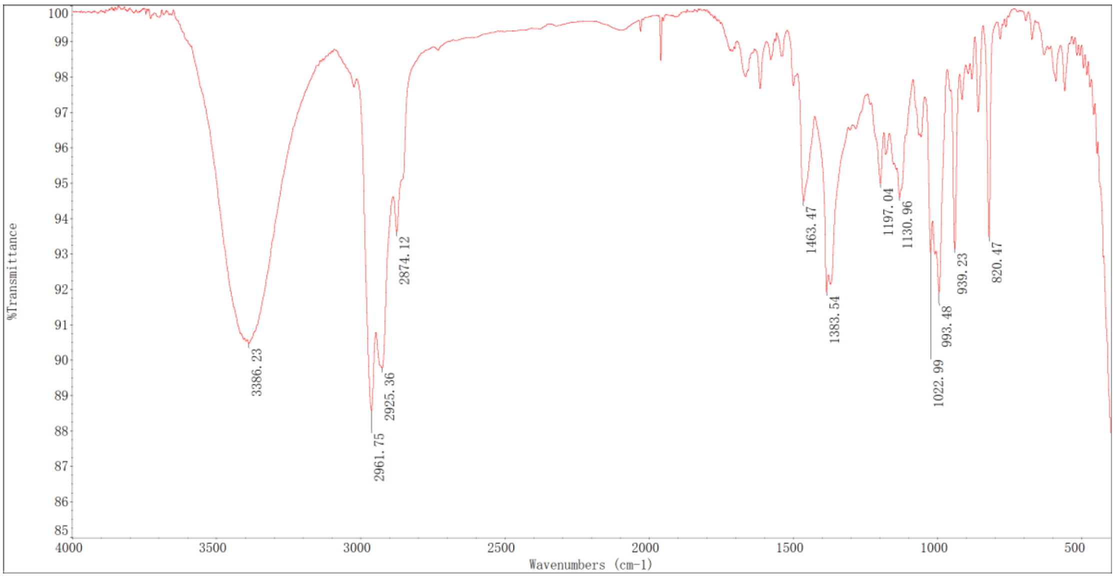

Figure S33. IR spectrum of **5**.

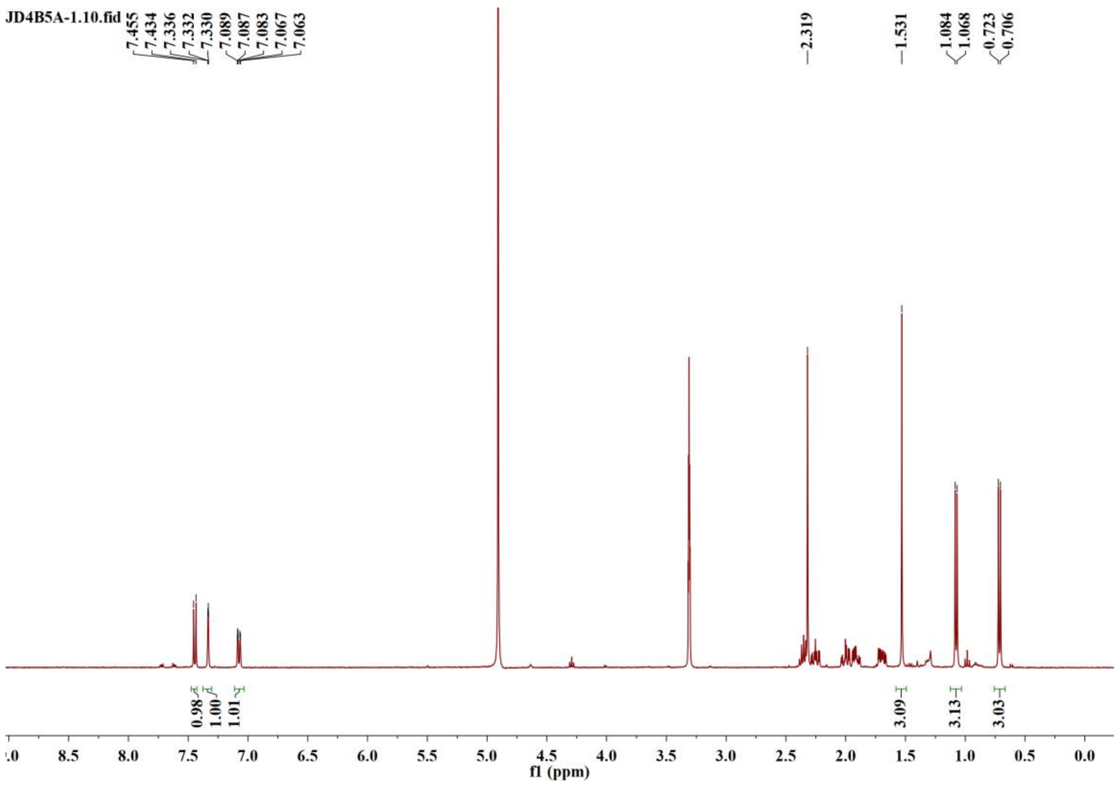

Figure S34. ^{1}H NMR spectrum (400 MHz) of (+)-**5** in CD_3OD.

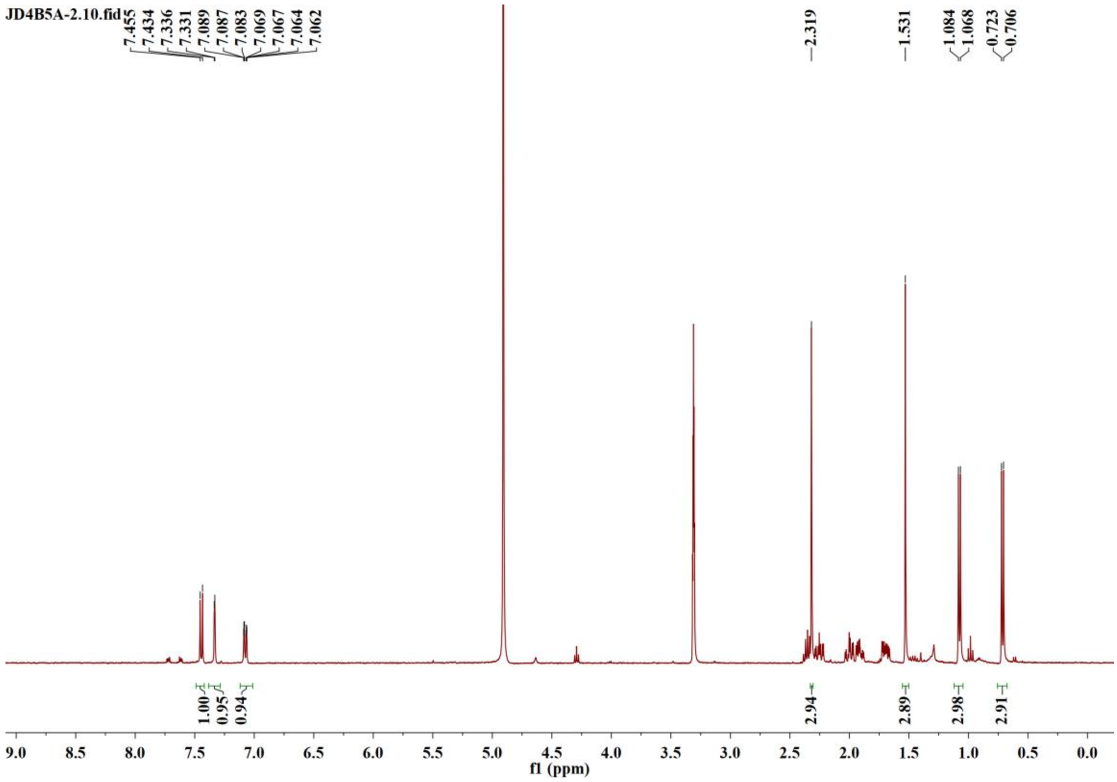

Figure S35. ^{1}H NMR spectrum (400 MHz) of (−)-**5** in CD_3OD.

5. TDDFT-ECD calculation of compound (+)-1.

5.1 Computational section.

Conformational searches were carried out using the torsional sampling (MCMM) method and OPLS_2005 force field. Conformers above 1% population were re-optimized at the B3LYP/6-311G(d,p) level of theory with IEFPCM (Polarizable Continuum Model using the Integral Equation Formalism variant) solvent model for acetonitrile. For the resulting geometries, ECD spectra were obtained by TDDFT calculations performed with the B3LYP/6-311G(d,p) level of theory with IEFPCM solvent model for acetonitrile. Finally, the Boltzmann-averaged ECD spectra were obtained with SpecDis1.62.

5.2 Computational data of (2*S*,4*S*,5*R*,6*S*,7*S*)-[(+)-**1**].

Torsional sampling (MCMM) conformational searches using OPLS_2005 force field were carried out by means of the conformational search module in the Macromodel1 applying an energy window of 21 kJ/mol, which afforded 4 conformers for (2*S*,4*S*,5*R*,6*S*,7*S*)-[(+)-**1**]. The Boltzmann populations of the conformers were obtained based on the potential energy provided by the MMFFs force field, giving 2 conformers for (2*S*,4*S*,5*R*,6*S*,7*S*)-[(+)-**1**] above 1% population for re-optimization. The re-optimization and the following TDDFT calculations of the re-optimized geometries (Fig. S36, Table S1) were all performed with Gaussian 09 at the B3LYP/6-311G(d,p) level with IEFPCM solvent model for acetonitrile. Frequency analysis was performed as well to confirm that the re-optimized geometries were at the energy minima. Finally, the SpecDis1.62 software was used to obtain the Boltzmann-averaged ECD spectra of (2*S*,4*S*,5*R*,6*S*,7*S*)-[(+)-**1**].

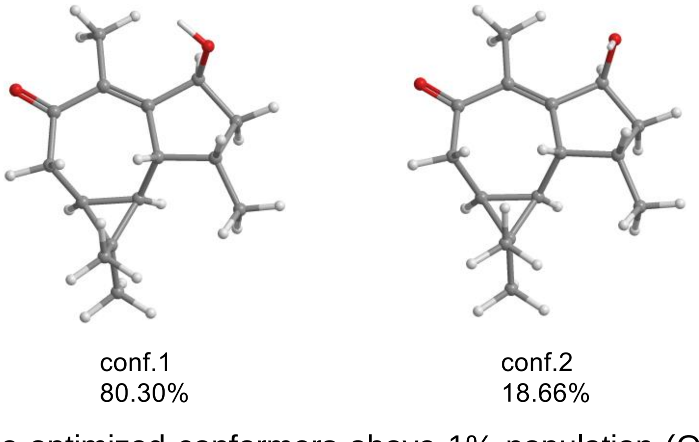

Figure S36. Re-optimized conformers above 1% population (OPLS_2005) of (2*S*,4*S*,5*R*,6*S*,7*S*)-[(+)-**1**] calculated at the B3LYP/6-311G(d,p) level of theory with IEFPCM solvent model for acetonitrile.

Table S1. Cartesian coordinates for the re-optimized conformers of (2*S*, 4*S*, 5*R*, 6*S*, 7*S*)-[(+)-**1**] at the B3LYP/6-311G(d,p) level of theory with IEFPCM solvent model for acetonitrile.

(2*S*,4*S*,5*R*,6*S*,7*S*)-[(+)-**1**] Conf. 1	Standard Orientation (Ångstroms)			
I	atom	X	Y	Z
1	C	1.25990900	0.18648400	0.02654900
2	C	-0.12807500	0.68897800	-0.37935400
3	C	-1.18193500	0.10330200	0.55657000
4	C	-1.42037000	-1.38409000	0.45942000
5	C	-0.62223300	-2.17151600	-0.56766000
6	C	0.84828800	-2.29679600	-0.18979200
7	C	1.69007400	-1.09370200	0.11371400
8	C	2.12908300	1.39560900	0.36861900
9	C	1.12224600	2.50526200	0.64998300
10	C	-0.00847200	2.24547500	-0.35807800
11	C	-2.53634700	-0.41257400	0.09680800
12	C	-1.29824300	3.01750700	-0.08795600
13	C	3.09954700	-1.45788900	0.52766500
14	C	-3.66810900	-0.30045900	1.10608700
15	C	-3.01762700	-0.25874700	-1.33498800
16	H	-0.34812300	0.37207900	-1.39955800
17	O	2.90675800	1.80235700	-0.77521800
18	O	1.35455800	-3.40977600	-0.11344700
19	H	-1.15650300	0.51384700	1.56219000
20	H	-1.55241300	-1.91604400	1.39755800
21	H	-1.00390000	-3.18969700	-0.66765600
22	H	-0.68312700	-1.70927300	-1.55603200
23	H	2.78957500	1.20930400	1.21959800
24	H	1.57848600	3.49262800	0.54857700
25	H	0.75269800	2.40854400	1.67710100
26	H	0.36689300	2.54318300	-1.34217200
27	H	-1.11100200	4.09442400	-0.13214900
28	H	-2.06614400	2.78417600	-0.83042100
29	H	-1.70686000	2.79093900	0.90038500
30	H	3.08604700	-2.12304100	1.39496100
31	H	3.59975000	-2.00913800	-0.27325500
32	H	3.70664500	-0.58839000	0.77252100
33	H	-4.42980800	-1.06627800	0.92412100
34	H	-3.30646100	-0.42386800	2.13004900
35	H	-4.15547800	0.67785600	1.03702200
36	H	-3.74274200	-1.04331300	-1.57576600
37	H	-3.52180700	0.70468300	-1.46231200
38	H	-2.22150800	-0.30972900	-2.07759100
39	H	3.42882600	1.04539000	-1.06337000

B3LYP/6-311G(d,p) Energy = -735.45333014 a.u.

(2*S*,4*S*,5*R*,6*S*,7*S*)-[(+)-**1**]<br>Conf. 2	Standard Orientation<br>(Ångstroms)			
I	atom	X	Y	Z
1	C	1.25398300	0.16943300	0.01380600
2	C	-0.12925000	0.68372000	-0.39005400
3	C	-1.18359800	0.12458400	0.56151000
4	C	-1.43475500	-1.36216100	0.47748100
5	C	-0.65850400	-2.16592300	-0.55511300
6	C	0.81881600	-2.30712300	-0.20624400
7	C	1.68004800	-1.11274900	0.07338400
8	C	2.12908900	1.36085500	0.41178300
9	C	1.12786600	2.49983600	0.62964500
10	C	0.01685900	2.23702000	-0.39866300
11	C	-2.54674700	-0.38401000	0.12009900
12	C	-1.26574200	3.03612400	-0.17784100
13	C	3.09570100	-1.46990800	0.46757300
14	C	-3.66565000	-0.25548300	1.14188000
15	C	-3.04416700	-0.23646300	-1.30674100
16	H	-0.36126600	0.35304500	-1.40314600
17	O	3.11862000	1.66965900	-0.58796700
18	O	1.30955300	-3.42693500	-0.12741300
19	H	-1.14325800	0.54313800	1.56340800
20	H	-1.56068300	-1.88662400	1.42060500
21	H	-1.05370900	-3.18039500	-0.63928700
22	H	-0.73252600	-1.71279800	-1.54688400
23	H	2.70844600	1.15286700	1.31120900
24	H	1.60475000	3.47627300	0.52032500
25	H	0.72040700	2.43735900	1.64576400
26	H	0.40851100	2.51045400	-1.38684500
27	H	-1.06034700	4.10850700	-0.24259000
28	H	-2.02063300	2.79621600	-0.93139800
29	H	-1.69821300	2.83859700	0.80656600
30	H	3.12122200	-1.94215700	1.45519300
31	H	3.50325100	-2.20175000	-0.23300800
32	H	3.74754800	-0.59990000	0.47194500
33	H	-4.43591400	-1.01582400	0.97348600
34	H	-3.29325200	-0.37588100	2.16232500
35	H	-4.14534200	0.72659400	1.07232100
36	H	-3.77518600	-1.01944100	-1.53450700
37	H	-3.54596500	0.72824000	-1.43333500
38	H	-2.25723200	-0.29470200	-2.05854500
39	H	2.68031400	1.70988100	-1.44558800

B3LYP/6-311G(d,p) Energy = -735.45290767 a.u.
